# American Ginseng Protein-Enriched Fraction Attenuates Neurobehavioral Deficits in MPTP-Induced Parkinson’s Disease Mice with Associated Modulation of Gut Microbiota and Metabolic Pathways

**DOI:** 10.3390/nu18203412

**Published:** 2026-10-18

**Authors:** Jie Li, Xu Yang, Jiahan Wang, Chunhui Yu, Jiahong Lu, Wenhui Lian, Wei Liu, Yu Zhao

**Affiliations:** 1 Jilin Ginseng Academy, Changchun University of Chinese Medicine, Changchun 130117, China; libaozi2333@126.com (J.L.); yangxu04@126.com (X.Y.); 16688200398@163.com (J.W.); chunhui_yu02@163.com (C.Y.); lujh@ccucm.edu.cn (J.L.); 2 College of Pharmacy of Pharmacy, Changchun University of Chinese Medicine, Changchun 130117, China; liuwei_cczyydx@163.com

**Keywords:** Parkinson’s disease, American ginseng protein-enriched fraction, gut microbiota, metabolomics, dietary supplementation

## Abstract

**Background/Objectives**: Parkinson’s disease (PD) currently lacks disease-modifying therapies. Available treatments offer only symptomatic relief and fail to halt progressive dopaminergic neuron loss. American ginseng (*Panax quinquefolius* L.) is a well-recognized functional food with documented neuroprotective properties, largely ascribed to its ginsenoside and polysaccharide fractions. However, its endogenous protein component, the American ginseng protein-enriched fraction (AGP), remains underexplored in the context of PD. This study aimed to investigate the neuroprotective effects of dietary AGP on PD and its association with the microbiota–gut–brain axis (MGBA). **Methods**: AGP was purified, structurally characterized, and then orally administered to male C57BL/6 mice with MPTP-induced PD for 28 days. Multi-omics analyses and Western blotting were employed to evaluate its efficacy and explore potential underlying mechanisms. **Results**: AGP treatment significantly improved motor function and place navigation performance in the Morris water maze, restored striatal dopamine levels, and reduced α-synuclein (α-Syn) aggregation in PD mice. In addition, AGP was associated with suppression of the TLR4/MyD88/NF-κB/ERK inflammatory pathway, the reshaping of the dysregulated gut microbiota composition, and the correction of metabolic disorders in the sphingolipid and arachidonic acid pathways. **Conclusions**: AGP was associated with significantly improved neuroprotective effects in PD mice, accompanied by concurrent modulations of the gut microbiota, metabolic profiles, and inflammatory signaling. These associations suggest potential MGBA involvement, although directionality requires further investigation. These findings support further investigation of AGP as a candidate dietary intervention for neurodegenerative diseases.

## 1. Introduction

Parkinson’s disease (PD), the second most common neurodegenerative illness globally, is becoming increasingly prevalent because of environmental factors and population ageing [1]. The progressive loss of dopaminergic neurons in the substantia nigra and the aberrant accumulation of α-synuclein (α-Syn), whose aggregation leads to Lewy body formation, are hallmarks of PD [2]. Clinically, patients typically exhibit non-motor symptoms like cognitive decline and autonomic dysfunction in addition to motor symptoms such as bradykinesia, muscle rigidity, resting tremor, and balance deficits [3]. The underlying mechanisms of PD involve several factors, including mitochondrial dysfunction, imbalances in protein homeostasis, increased oxidative stress, and the activation of neuroinflammatory pathways [4]. Levodopa and dopamine receptor agonists, which successfully reduce motor symptoms but do not stop disease progression, are currently the most common therapeutic options [5]. Furthermore, the rate of missed diagnoses in early-stage PD remains high, resulting in lost opportunities for timely intervention [6]. Therefore, it is crucial to explore new mechanisms of this disease, such as the control of the microbiota–gut–brain axis (MGBA), and to develop innovative interventions that could delay disease progression and overcome existing treatment limitations. In this context, dietary bioactive proteins from natural plant sources have emerged as promising nutritional strategies for adjunctive management of neurodegenerative conditions, yet their specific efficacy in PD remains largely unexplored.

The pathophysiology of PD is significantly influenced by metabolic disorders, which are characterized by broad abnormalities across numerous pathways, involving lipid, amino acid, and energy metabolism [7]. Among these, lipid metabolism disorders are particularly important. According to clinical research, PD patients’ blood levels of sphingolipids and lysophosphatidylcholine are significantly elevated, suggesting the aberrant activation of the sphingolipid metabolic pathway [8,9,10]. The underlying pathophysiology of PD is intimately linked to this metabolic imbalance. For example, mutations in the *GBA1* gene result in decreased glucocerebrosidase activity, which causes an aberrant sphingolipid build-up in neurons. This build-up leads to the formation of Lewy bodies and promotes α-Syn misfolding and aggregation [8]. The development of PD is also significantly influenced by an imbalance in polyunsaturated fatty acids (PUFAs) in the brain. In particular, metabolic pathways involving arachidonic acid (AA) and linoleic acid are altered, and the ratio of pro-inflammatory n-6 PUFAs to anti-inflammatory n-3 PUFAs becomes imbalanced. Together, these elements worsen oxidative stress and neuroinflammation, hastening the death of dopaminergic neurons [11]. These metabolic insights not only elucidate novel mechanisms underlying PD pathogenesis but also offer key clues to understanding pathological gut–brain connections in this disease.

The MGBA has recently received considerable attention in the study of PD, especially in relation to how it interacts with metabolic dysregulation. There is growing evidence that changes in the composition of the gut microbiota are strongly linked to the degeneration of dopaminergic neurons, central nervous system (CNS) inflammation and MGBA dysfunction. These pathological features highlight the need for in-depth research in this area [12,13]. The intestinal microbiota of PD patients exhibits unique features, most notably a marked reduction in the synthesis of short-chain fatty acids (SCFAs) [11]. These fatty acids have anti-inflammatory properties and are crucial for maintaining the stability of the intestinal barrier. This alteration aligns closely with the “enteric origin” hypothesis proposed by Braak, which posits that the pathological processes associated with PD may initiate in the enteric nervous system [11,12,13,14]. Further studies have demonstrated that an imbalance in the intestinal microbiota can trigger an inflammatory response in intestinal glial cells. This response promotes the misfolding and aggregation of α-Syn within the intestine [12,14]. Researchers have used specially engineered animal models that express toxic α-Syn in the intestines to directly demonstrate that these abnormal proteins can travel retrogradely along the vagus nerve to the brainstem and then spread throughout the CNS. This process ultimately leads to neuronal loss and motor impairments. Notably, in vivo imaging studies have shown that α-Syn aggregates appear in the gut prior to the brain, providing strong support for the “enteric origin” theory [12,13,15]. These results imply that central metabolic homeostasis might be associated with an imbalance in the gut microbiota and changes in its metabolites from an MGBA perspective. This disruption can lead to protein misfolding and neuroinflammation, ultimately resulting in the characteristic neuronal degeneration observed in PD. Therefore, intervention strategies that target the MGBA may not only improve the metabolic environment by regulating the gut microbial ecosystem, but also address the underlying processes of PD, laying the groundwork for the development of new drugs with disease intervention effects.

American ginseng (*Panax quinquefolius* L.) is traditionally used in conventional medicine and as a dietary supplement. Ginsenosides, polysaccharides, and proteins are among its many bioactive components and contribute to its primary health benefits. However, current research focuses primarily on small molecules such as ginsenosides, while the biological activities of American ginseng protein-enriched fraction (AGP) remain underexplored [16,17]. Previous research has demonstrated that AGP can increase the activity of antioxidant enzymes such as superoxide dismutase (SOD) and glutathione peroxidase (GSH-Px), lower the levels of oxidative stress markers such as malondialdehyde (MDA), and regulate important factors involved in mitochondrial biogenesis in mice, such as nuclear respiratory factor 1 (NRF-1) and mitochondrial transcription factor A (TFAM) [18]. These functions align closely with the core pathological features of PD, which include mitochondrial malfunction and oxidative stress. Additionally, an imbalance in the gut microbiota can result in both peripheral and cerebral inflammatory responses, and new research has demonstrated a clear correlation between MGBA malfunction and the onset of PD. Parallel to these findings, the regulatory capacity of plant-derived protein components in maintaining intestinal ecosystem balance has been increasingly recognized [19,20]. Given the central role of oxidative stress in PD pathogenesis and the key regulatory role of the MGBA in disease progression, we speculate that AGP may have considerable yet underexplored potential for PD intervention, due to its inherent antioxidant capacity and ability to regulate intestinal microbiota homeostasis [21].

However, few systematic studies have investigated the anti-PD effects of AGP, especially how AGP regulates dopaminergic neurons in the substantia nigra pars compacta (SNpc). To address this research gap, in the present study, we first purified and comprehensively characterized AGP derived from *Panax quinquefolius* L., and subsequently evaluated its protective effects on neuroinflammation, motor dysfunction, and the loss of nigral dopaminergic neurons in an MPTP-induced mouse model of PD. Untargeted metabolomics and 16S rRNA gene sequencing of the gut microbiota were further integrated to profile the alterations in serum and fecal metabolites, as well as the reshaping of gut microbial community structure after AGP intervention. Western blot analysis was subsequently conducted to examine the core molecular mechanism, which indicated a potential regulatory association of AGP with neuroinflammation through the MyD88-TLR4-NF-κB signaling cascade and the ERK signaling pathway. From a nutritional perspective, this study frames AGP as a naturally occurring dietary bioactive protein rather than a conventional synthetic pharmacological agent, suggesting its potential as a candidate for further investigation in daily intervention for PD. By combining macroscopic behavioral assessment, microscopic histological observation and multiomics molecular verification, this work systematically explores the potential mechanisms of AGP in relation to PD across multiple levels, and offers comprehensive evidence in favor of further exploration of AGP in the nutritional treatment of PD.

## 2. Materials and Methods

### 2.1. Materials and Reagents

The dried roots of American ginseng were obtained from a certified local supplier and authenticated by Prof. Dacheng Jian at the Changchun University of Traditional Chinese Medicine (Changchun, China). Sigma-Aldrich Corporation (St. Louis, MO, USA) provided levodopa, carbidopa, and MPTP chemical reagents. The Sustained-release Tablets Carbidopa and Levodopa were obtained from Merck & Co., Inc. (Kenilworth, NJ, USA). Shanghai Enzyme-Linked Biotechnology Co., Ltd. (Shanghai, China) provided interleukin-1β (IL-1β), interleukin-10 (IL-10), TNF-α, total antioxidant capacity (T-AOC), SOD, GSH-Px, and MDA detection kits. Tyrosine Hydroxylase (TH, ab137869), α-Syn (ab138501), Toll-like receptor 4 (TLR4), nuclear factor-κB p65 (NF-κB p65), phospho-NF-κB p65 (p-p65), extracellular signal-regulated kinase (ERK), phospho-ERK (p-ERK), β-tubulin and glyceraldehyde-3-phosphate dehydrogenase (GAPDH) were purchased from Cell Signaling Technology (Danvers, MA, USA) or Abcam (Cambridge, UK). All remaining reagents were of analytic grade.

### 2.2. Purification and Characterization of AGP

The dried American ginseng root powder (100 g) was extracted three times sequentially with 0.1 M NaOH and deionized water adjusted to pH 7.0. After centrifugation (10,000× *g*, 20 min, 4 °C), the combined supernatant and was acidified with 1 M HCl to pH 3.5 and the protein was precipitated at 4 °C for 12 h. To exclude small-molecule contaminants, the precipitate was recovered by re-centrifugation (12,000× *g*, 30 min, 4 °C), re-dissolved in PBS buffer (pH 7.0), and ultrafiltered through a 3 kDa molecular weight cut-off (MWCO) membrane (Millipore, Burlington, MA, USA) at 4500 rpm for 60 min. The retained fraction was collected and lyophilized to obtain purified AGP powder and kept at −20 °C until required. The extraction yield of lyophilized AGP powder was 3.05% ± 0.06% (*w*/*w*) from dried American ginseng root. The protein content of the lyophilized AGP powder was determined using the Bradford assay, and the purity of the powder (81.42% ± 1.08%) was calculated as the percentage of protein content relative to the total dry weight of the powder. The polysaccharide content was 18.40% ± 0.91%, and protein and polysaccharide contents together accounted for 99.82% of the lyophilized powder, corresponding to an AGP protein-enriched fraction yield of approximately 2.48 g per 100 g of dried root. The supporting information provides detailed structural characteristics, including SDS-PAGE data and amino acid composition.

### 2.3. Animals and Treatments

C57BL/6 male specific pathogen-free (SPF) mice, 10 weeks old (26–28 g) were purchased from Changchun Yishi Laboratory Animal Technology Co., Ltd. (Changchun, China). All animals were kept in controlled environments (20–25 °C, 50–65% relative humidity, 12 h light/dark cycles) with unlimited access to water and standard rodent food. Mice were housed in multiple cages per group. Cage was not included as a random effect in the statistical models, and potential cage effects cannot be fully excluded. After a week of acclimatization, 60 mice were randomly assigned to six groups (*n* = 10) using a computer-generated random number sequence: Control, Model (MPTP), Positive Control (P.C., treated with 70 mg/kg Carbidopa–levodopa), and three AGP intervention groups receiving oral AGP in low doses (AGPL, 40 mg/kg), medium doses (AGPM, 80 mg/kg), and high doses (AGPH, 120 mg/kg). We set 10 animals per group to accommodate multiple experiments (each requiring different samples), compensate for MPTP-induced mortality, and meet the standard 6–10 replicates for metabolic and microbiological analyses, ensuring robust statistical performance and representativeness. The AGP dosages were selected based on the effective doses established in our previous study on ginseng protein in MPTP-induced PD mice. During the AGP treatment period, working solutions were freshly prepared each week. The protein concentration was adjusted according to the mean body weight of each group to maintain the target doses, using a fixed gavage volume of 0.2 mL per mouse. Working solution concentrations were expressed as AGP protein-enriched fraction protein. The mean body weights and corresponding working solution protein concentrations for each group are provided in Supplementary Table S2. The approximate human equivalent doses (HEDs) for mouse doses of 40, 80, and 120 mg/kg are 3.2, 6.5, and 9.7 mg/kg/day, corresponding to approximately 195, 389, and 584 mg/day for a 60 kg adult. These values refer to AGP and require validation in human trials. All groups, except the Control group, received intraperitoneal injections of MPTP (35 mg/kg) every other day for 28 days to construct the PD model [22].

After the model was performed, 12 mice died or were euthanized due to severe toxicity: 3 in the Model group, 2 in the P.C. group, 2 in the AGPL group, 3 in the AGPM group, and 2 in the AGPH group; no deaths occurred in the Control group. These losses occurred mainly in the early stage and were attributable to the acute toxicity of MPTP, which is consistent with previous reports using similar chronic regimens [23]. Humane euthanasia was immediately performed on mice that reached any of the following pre-specified humane endpoints: a body weight reduction of ≥20% relative to baseline, persistent immobility for more than 24 consecutive hours, total absence of voluntary food and water consumption. On the other hand, the Control group received equivalent quantities of sterile saline. After modeling, the P.C. group received oral Carbidopa–levodopa (70 mg/kg/day), the three AGP groups received their respective oral doses of AGP, and the Control and Model groups received saline by gavage for an additional 28 days (designed to mimic a dietary supplementation regimen). During the experiment, body weight was recorded every seven days, and behavioral tests were carried out after the final administration. The final sample sizes for behavioral analyses were: Control *n* = 10, Model *n* = 7, P.C. *n* = 8, AGPL *n* = 8, AGPM *n* = 7, and AGPH *n* = 8. In gut microbiome analysis (16S rRNA sequencing) and untargeted metabolomics (serum and fecal UPLC-Q Exactive MS), six mice per group were randomly selected from the surviving animals. For serum inflammatory cytokine measurements, histology, immunofluorescence, brain oxidative stress markers (MDA, SOD, GSH-Px, T-AOC), and Western blotting, 3 mice per group were randomly selected due to the higher cost and more labor-intensive tissue/serum processing requirements. Random selection was performed using computer-generated random number sequences. All outcome assessments were independently performed by investigators who remained fully blinded to group allocation throughout the study. After the last behavioral tests, blood was collected by retroorbital puncture, and the mice were humanely euthanized by CO_2_ inhalation. Brain tissues (striatum and substantia nigra) and colon tissues were rapidly dissected, frozen in liquid nitrogen, and stored at −80 °C for further analysis. All experimental protocols were approved by the Experimental Animal Ethics Committee of the Changchun University of Traditional Chinese Medicine (Approval No. 2025923) and strictly followed the ARRIVE guidelines.

### 2.4. Behavioral Tests

#### 2.4.1. Suspension Rope Test

Grip strength and motor coordination were evaluated by a wire suspension test [22]. The mice were suspended from a horizontal rope (1 cm in diameter and 30 cm in height) fixed at both ends and the grasp time was recorded. Performance was evaluated on a scale of 0–4 based on the use of the limbs: 4 points (both hindlimbs), 3 points (one hindlimb), 2 points (both forelimbs), 1 point (one forelimb), and 0 points (failure to grasp). Each mouse was subjected to three formal 2-min trials after three days of acclimatization, and the average score was determined.

#### 2.4.2. Rotating Rod Test

To evaluate motor strength and balance coordination, the rotarod test was used [23]. The mice were placed on a rotating rod that accelerated from 15 to 40 rpm, and the latency to fall onto a padded platform below was recorded in seconds. To ensure reliability, mice received three days of standardized training (starting at 15 rpm and increasing by 5 rpm daily). For formal tests, each mouse completed the test with a 15 min intertrial interval, and statistical analyses were performed using the average fall latency.

#### 2.4.3. Morris Water Maze

The Morris water maze was used to assess place navigation performance [24]. A computerized video tracking system (ANY-maze, Stoelting Co., Wood Dale, IL, USA) was used to capture the movement trajectories in a darkly illuminated room with a circular pool (1.2 m in diameter, 20 cm in water depth, 22 ± 1 °C). A visible platform (1 cm above the water surface) was placed in a fixed quadrant for 7 days of training. Mice were released from the start position in a randomized manner and allowed to locate the platform for up to 120 s; if they failed, they were gently directed to it and allowed to remain there for 10 s. The platform was immersed in water 1–2 cm below the surface on the test day and titanium dioxide (TiO_2_) was added to make the water opaque. For each mouse, escape latency, swimming distance, and path length were recorded.

### 2.5. Hematoxylin and Eosin (H&E) Staining

Brain tissues were fixed in 4% paraformaldehyde, embedded in paraffin, and sectioned coronally through the substantia nigra and hippocampus regions at a thickness of 4 μm. Sections were dewaxed and rehydrated, and H&E dyeing, dehydration, xylene cleaning, and neutral balsam sealing were carried out. A light microscope (Olympus BX53, Tokyo, Japan) was used to assess histopathological changes.

### 2.6. Immunofluorescence

Brain sections (4 μm thick) were blocked with 5% normal goat serum after being fixed and permeabilized with 0.1% Triton X-100. Subsequently, they were incubated overnight at 4 °C with primary antibodies α-Syn (1:1,000, ab138501, Abcam) and TH (1:200, ab137869, Abcam). The sections were washed and then incubated for 1 h at room temperature using secondary antibodies labeled with Alexa Fluor^®^ 488/594 (1:500, Invitrogen, Carlsbad, CA, USA). DAPI was used as a nuclear counterstain. A fluorescence microscope (Nikon Eclipse Ti2, Tokyo, Japan) was used to observe and photograph immunofluorescent signals, and the ImageJ software (version 1.54p, NIH, Bethesda, MD, USA) was utilized to measure the integrated optical density (IOD) of the positive area.

### 2.7. Immunohistochemistry

Paraffin-embedded sections containing the substantia nigra were deparaffinized and exposed to a citrate buffer (pH 6.0) for the recovery of antigen. The endogenous peroxidase activity was eliminated with 3% H_2_O_2_. After blocking with 5% goat serum for 1 h at room temperature, the sections were incubated overnight at 4 °C with primary antibodies Iba1 (1:2,000, Wako, Osaka, Japan) and GFAP (1:1,000, Abcam, Cambridge, UK). After washing with PBS, the sections were incubated for 30 min with a secondary antibody conjugated with HRP (1:2000, Cell Signaling Technology, Danvers, MA, USA). Immunoreactivity was visualized with 3,3′-diaminobenzidine (DAB) substrate, and the sections were counterstained with hematoxylin. Immunopositive cells were imaged and quantified by ImageJ software.

### 2.8. Detection of Neurotransmitter Levels via ELISA

Samples of striatal tissue were homogenized in 0.9% ice-cold saline (*w*/*v*). After centrifuging the homogenates at 3,000× *g* for 10 min at 4 °C, the supernatants were collected. The levels of dopamine (DA), homovanillic acid (HVA), and dihydroxyphenylacetic acid (DOPAC) were measured using commercially available ELISA kits (Shanghai Enzyme-Linked Biotechnology Co., Ltd., Shanghai, China). A microplate reader (BioTek Epoch, Winooski, VT, USA) was used to measure the absorbance at 450 nm.

### 2.9. Measurement of Biochemical Indices

Protease inhibitors (Roche, Basel, Switzerland) were added to ice-cold RIPA lysis solution, and brain tissues (substantia nigra) were homogenized in this solution. The supernatants were collected by centrifugation (10,000× *g*, 10 min, 4 °C). Commercial assay kits (Nanjing Jiancheng Bioengineering Institute, Nanjing, China) were used to measure T-AOC, SOD, GSH-Px, and MDA levels according to the manufacturer’s instructions. Whole blood was clotted for 1.5 h at room temperature and centrifuged (12,000× *g*, 10 min, 4 °C) to extract serum for inflammatory cytokines analysis. Specific ELISA kits were used to measure the levels of TNF-α, IL-1β, and IL-10 (Shanghai Enzyme-Linked Biotechnology Co., Ltd., Shanghai, China).

### 2.10. UPLC-Q Exactive MS Analysis of Serum and Fecal Metabolites

Matrix-specific procedures were used to recover metabolites from serum and feces. For serum, an equal volume of methanol was added to 100 µL aliquots. The mixture was then centrifuged (12,000× *g*, 10 min, 4 °C), dried under a nitrogen stream, and reconstituted in acetonitrile/water (1:1, *v*/*v*). For feces, 1,200 µL ice-cold methanol was used to extract 300 mg of powder sample, which were then vortexed, centrifuged (14,000× *g*, 10 min, 4 °C), ultrasonicated (20 min, 4 °C), and filtered through a 0.22 µm membrane. To evaluate system stability, quality control (QC) samples were prepared by combining equal amounts from each sample and injected every ten cycles.

An ultra-high-performance liquid chromatography (UHPLC) system (Thermo Fisher Scientific, Waltham, MA, USA) connected to a Q Exactive Orbitrap mass spectrometer was used for chromatographic separation. A Thermo Scientific Syncronis C18 column (2.1 × 100 mm, 1.7 µm) was used to separate serum at 30 °C with a mobile phase consisting of acetonitrile (B) and 0.1% formic acid in water (C) at 0.4 mL/min. The gradient was: 0–4 min (5–30% B), 4–7 min (30–50% B), 7–11 min (50–70% B), 11–14 min (70–80% B), 14–15 min (80% B), 15–18 min (80–40% B), 18–19 min (40–5% B), and 19–20 min (5% B). The Waters ACQUITY UPLC^®^ HSS T3 column (2.1 × 150 mm, 1.8 µm, Waters Corporation, Milford, MA, USA) with a modified gradient was used for feces: 0–2 min (5–30% B), 2–4 min (30–60% B), 4–6 min (60% B), 6–7 min (60–90% B), 7–13 min (90–5% B), and 13–15 min (5% B). Positive and negative electrospray ionization (ESI) modes were used in mass spectrometry, with scan ranges of *m*/*z* 150–1500 in serum and *m*/*z* 100–1500 in feces. The common parameters were as follows: Resolution 70,000 (FWHM), AGC target 1 × 10^6^ ions, sheath gas 40 arb, auxiliary gas 10 arb, spray voltage 4.0 kV, capillary temperature 350 °C, and S-lens RF level 55.

Compound Discoverer 3.0 (Thermo Fisher Scientific, Waltham, MA, USA) was used to process data (peak detection, alignment, and deconvolution). For principal component analysis (PCA) and orthogonal partial least-squares discriminant analysis (OPLS-DA), the resulting matrix (retention time, *m*/*z*, and peak area) was imported into SIMCA-P 14.1 (Umetrics, Umeå, Sweden). The differential metabolites of the serum were screened using VIP > 1.0, *p* < 0.05, |FC| > 1.2, and Benjamini–Hochberg FDR correction (q < 0.15). Fecal metabolites were screened using the same criteria (VIP > 1.0, |FC| > 1.2), with Benjamini–Hochberg FDR correction at q < 0.25. These fecal results are presented as exploratory, as the primary focus of this study is on serum metabolites for systemic metabolic profiling and correlation analyses. The MS/MS fragments were compared with the Human Metabolome Database (HMDB, v5.0), and putative identifications were made. Each potential metabolite was assigned a level of confidence consistent with the Metabolomics Standards Initiative (MSI) guidelines. All metabolites reported in this study were presumed to be identified at MSI level 2 (by MS/MS spectral matching to the Human Metabolome Database, HMDB v5.0). To minimize the risk of false annotations, we manually checked all MS/MS spectra and excluded features with poor spectral matching or those likely originating from environmental contaminants (e.g., plasticizers, detergents) or artificial source fragmentation. MetaboAnalyst 6.0 (Xia Lab, McGill University, Montreal, QC, Canada)was used for pathway enrichment and heat map visualization.

### 2.11. 16S rRNA Sequencing of the Gut Microbiota

The total microbial DNA was extracted from fecal samples (0.3 g) using the E.Z.N.A.^®^ Stool DNA Kit (Omega Biotek, Norcross, GA, USA). The 338F (5′-ACTCCTACGGGAGGCAGCAG-3′) and 806R (5′-GGACTACHVGGGTWTCTAAT-3′) primers were used to amplify the V3–V4 hypervariable regions of the16S rRNA gene of bacteria. Purified amplicons were pooled in equimolar amounts and sequenced on an Illumina NovaSeq PE250 platform (Illumina, San Diego, CA, USA) and produced 2 × 250 bp paired end reads. QIIME 2 (v2022.2) was used for demultiplexing and quality filtering. Low-quality reads (quality score < 20) were trimmed, and sequences shorter than 200 bp were removed. Chimeric sequences were identified and removed using the VSEARCH (version 2.13.4) software. Due to the higher accuracy and resolution of amplicon sequence variant (ASV) inference than traditional operational taxonomic unit (OTU) clustering, the DADA2 pipeline was used for denoising, primer trimming, paired-end merging, and chimera removal. Singleton ASVs (those with a total count of one across all samples) were removed to eliminate spurious artifacts, yielding high-quality representative ASV sequences and ASV abundance tables for downstream analyses. To reduce bias from uneven sequencing depth and enable diversity comparisons among groups, rarefaction was performed before alpha and beta diversity analyses. All valid samples were rarefied to 95% of the minimum sequencing depth, ensuring sufficient coverage while retaining maximal valid biological information. Rarefaction curves confirmed adequate sequencing depth and saturation of microbial diversity. The Greengenes database (v13.8) was used for taxonomic assignment. In accordance with the Greengenes/GTDB taxonomic nomenclature, genus-level labels containing alphabetic suffixes indicate that polyphyletic taxa require differentiation to reflect phylogenetic relationships. Labels with ‘unclassified’ indicate that classification cannot assign the sequence to a lower taxonomic ranking at the specified confidence threshold. The Genes Cloud platform (https://www.genescloud.cn) was employed to evaluate alpha diversity (Chao1, Good’s coverage, Simpson, and Pielou’s evenness) and beta diversity based on Bray–Curtis distance matrices. Principal coordinate analysis (PCoA) and non-metric multidimensional scaling (NMDS) were carried out to visualize the structure of the microbial community, with ellipses representing 95% confidence intervals for each group. PERMANOVA (permutational multivariate analysis of variance) with 999 permutations was used to test significant differences in beta diversity between groups. LEfSe (Linear Discriminant Analysis Effect Size) was performed to identify differentially abundant genera across groups, with the factorial Kruskal–Wallis test set at an alpha value of 0.05, a logarithmic LDA score threshold > 2.0, and a one-against-all comparison strategy. Spearman’s rank correlation analyses were conducted to assess associations among differentially abundant genera, metabolites, and phenotypic indicators, with cut-off criteria defined as |r| > 0.6 and Benjamini–Hochberg FDR < 0.05.

### 2.12. Western Blotting

Total protein was extracted from the SNpc using RIPA buffer containing protease and phosphatase inhibitor cocktails (Roche Diagnostics, Mannheim, Germany). The BCA assay kit (BioRay Biotechnology, Shanghai, China) was used to measure protein concentrations. The same amount of protein was separated by 10% SDS-PAGE and then transferred to PVDF membranes (Merck Millipore, Burlington, MA, USA). The membranes were blocked for 1 h at room temperature with 5% skim milk and then incubated overnight at 4 °C with primary antibodies against TLR4 (1:1,000), MyD88 (1:1,000), NF-κB p65 (1:1,000), phospho-NF-κB p65 (p-p65, 1:1,000), ERK (1:2,000), and phospho-ERK (p-ERK, 1:2,000). After washing with TBST, membranes were incubated for 1 h with secondary antibodies conjugated with HRP at room temperature (1:5,000, Cell Signaling Technology, Danvers, MA, USA). Protein bands were observed with enhanced chemiluminescence (ECL, BioRay Biotechnology, Shanghai, China) and recorded with a ChemiDoc XRS imaging system (Bio-Rad, Hercules, CA, USA). ImageJ was used to quantify band intensities, which were normalized to the corresponding loading control (β-tubulin or GAPDH). Three independent biological replicates were carried out. In Western blot analysis, three animals were randomly selected from each experimental group and used as biological replicates (*n* = 3 mice per group).

### 2.13. Statistical Analyses

R software (v4.1.0), GraphPad Prism 8.0.2 (GraphPad Software, San Diego, CA, USA), and SPSS 19.0 (IBM, Armonk, NY, USA) were used for statistical analysis. Normality was assessed using the Shapiro–Wilk test, and the homogeneity of variances was verified using the Levene test. For body weight, data were collected repeatedly in the course of experiments, and repeated ANOVA was carried out in both directions with group and time as factors, followed by Bonferroni’s post hoc test; sphericity was verified using Mauchly’s test, and if violated, Greenhouse–Geisser correction was applied. Body weight data are presented as mean ± SEM. All other data were analyzed using one-way ANOVA and Dunnett‘s post hoc test to compare each treatment group to the Model group, and presented as mean ± SD. The threshold for statistical significance was set at *p* < 0.05. For microbiota data, α-diversity, β-diversity, and Spearman correlation matrices were generated using the Genes Cloud platform, and co-occurrence network analysis was performed in R with cut-off criteria of |r| > 0.6 and Benjamini–Hochberg FDR < 0.05. All schematic mechanism diagrams were constructed using the online scientific illustration platform Figdraw (http://www.figdraw.com).

## 3. Results

### 3.1. AGP Supplementation Alleviates Behavioral Abnormalities in PD Mice

**Figure 1:**
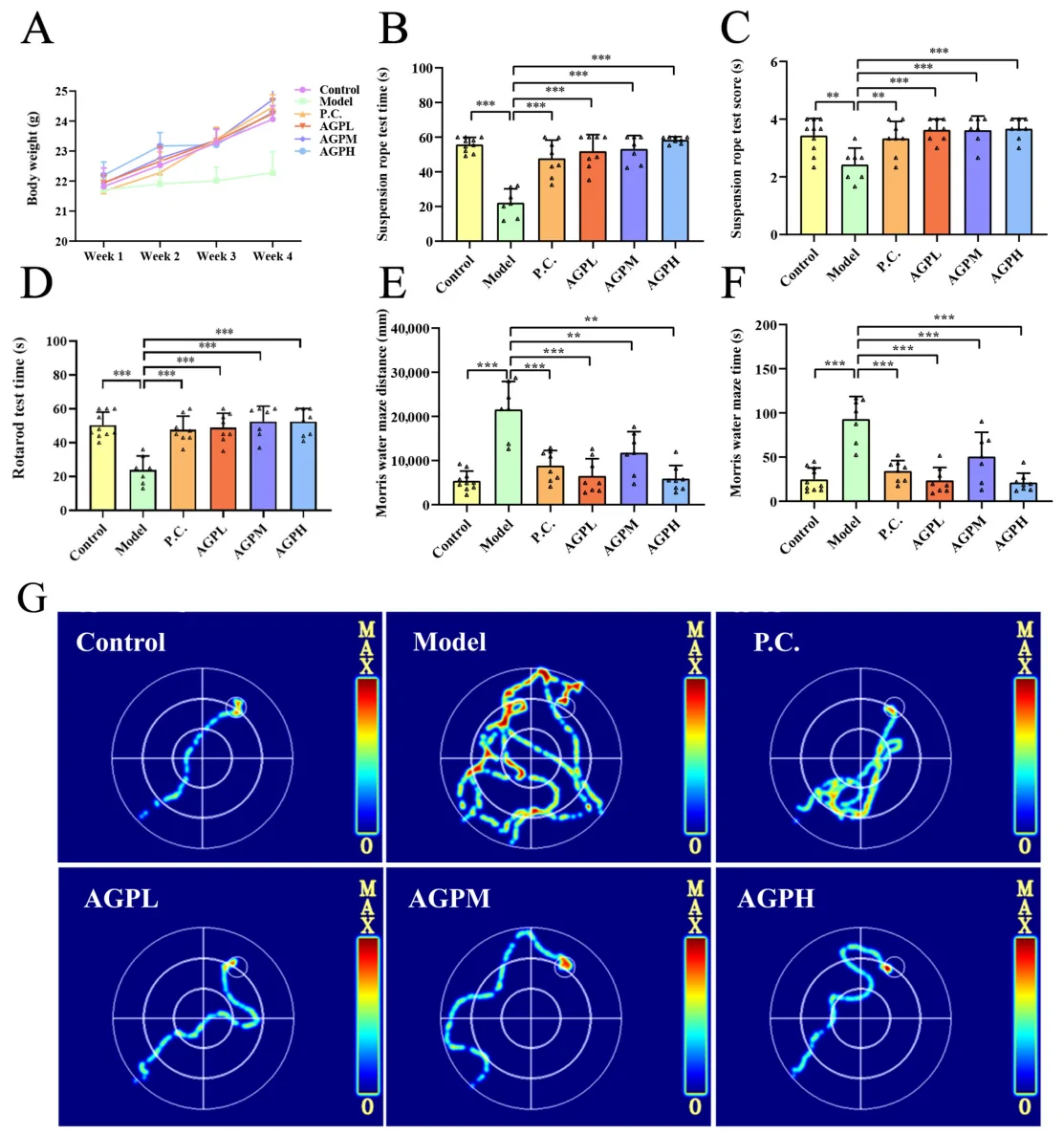
Effects of dietary AGP supplementation on motor function in MPTP-induced PD mice. (**A**) Body weight changes over the experimental period. (**B**) Suspension rope test time. (**C**) Suspension rope test score. (**D**) Latency to fall in the rotarod test. (**E**) Total swimming distance in the Morris water maze. (**F**) Escape latency in the Morris water maze. (**G**) Representative swimming trajectories during the Morris water maze test. Data are presented as mean ± SD. All surviving animals underwent behavioral testing and were included in the behavioral statistical analyses in Figure 1: Control (*n* = 10), Model (*n* = 7), P.C. (*n* = 8), AGPL (*n* = 8), AGPM (*n* = 7), and AGPH (*n* = 8). ** *p* < 0.01, *** *p* < 0.001.

To systematically evaluate the effects of AGP intervention as an oral food supplement on MPTP-induced PD model mice, with MPTP-induced mortality and per-mouse usage detailed in Figure S1, we conducted a series of behavioral tests. The body weight was recorded every seven days during the experiment. All groups showed a steady increase in body weight during the study, as shown in Figure 1A. Two-way repeated-measures ANOVA revealed a significant main effect of time (*p* < 0.0001), but no significant main effect of group (*p* = 0.3290) and no significant time × group interaction (*p* = 0.6453). Bonferroni’s post hoc test showed no significant differences between the Model group and any other group at any time point (all adjusted *p* > 0.05). Nevertheless, mice treated with AGP consistently showed higher average body weight than model mice from Week 2 onward, suggesting a trend toward reducing MPTP-induced growth delays. The suspension rope test was performed to assess the limb strength and endurance (Figure 1B,C). Compared to the Control group, MPTP model mice displayed significantly shorter hanging times and lower hanging wire scores (*p* < 0.001, *p* < 0.01). Notably, oral administration of AGP significantly prolonged the suspension rope test time and restored the suspension rope test score (*p* < 0.001), indicating a significant improvement in limb strength and muscle endurance. The rotarod test was used to assess the balance and motor coordination (Figure 1D). Compared to control mice, MPTP model mice spent considerably less time on the rotating rod (*p* < 0.001). Importantly, AGP-supplemented groups showed significantly longer falling latencies than the model group (*p* < 0.001), suggesting that AGP effectively improved the motor coordination deficit in PD mice. The Morris water maze test was used to assess place navigation performance (Figure 1E–G). MPTP model mice exhibited significantly longer escape latencies and greater total swimming distances than control mice (*p* < 0.001). AGP supplementation significantly reduced both parameters, suggesting improved place navigation performance in AGP-treated mice. Swimming velocity did not differ significantly among the groups (Supplementary Figure S3). However, because motor impairment cannot be completely excluded, escape latency and swimming distance were interpreted primarily as measures of place navigation performance rather than as pure cognitive measures. Collectively, these findings suggest that AGP, when administered as a dietary supplement, exerts multi-dimensional protective effects on MPTP-induced PD model mice, effectively alleviating pathological phenotypes associated with impaired weight gain, motor dysfunction, and deficits in place navigation performance.

### 3.2. AGP Supplementation Reduces Neuronal Damage in PD Model Brains

**Figure 2:**
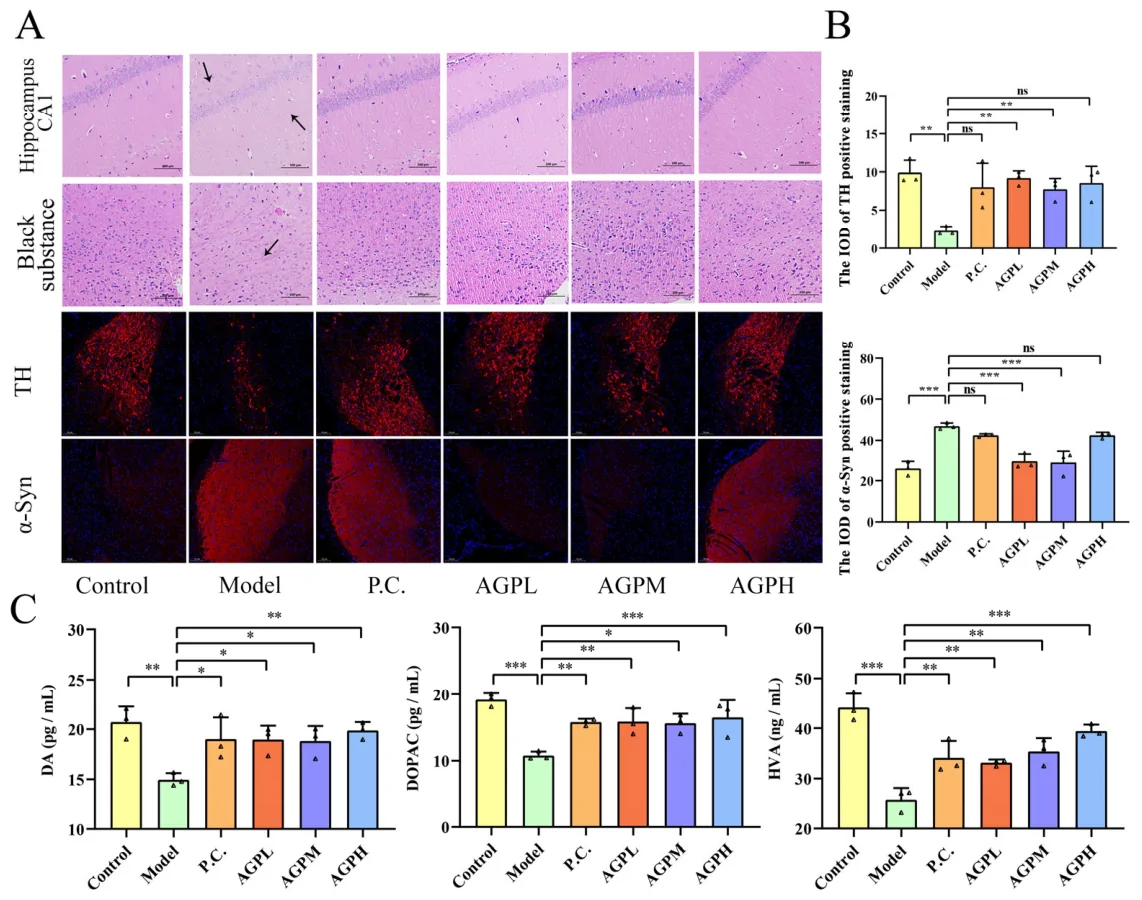
Dietary AGP supplementation protects against MPTP-induced neuropathology and dopaminergic dysfunction in PD mice. (**A**) H&E and immunofluorescence in hippocampus and SNpc. (**B**) α-Syn and TH immunofluorescence in SNpc. (**C**) Striatal DA, DOPAC, and HVA levels. Data are presented as mean ± SD (*n* = 3 per group for immunofluorescence and neurotransmitter analyses). ns, not significant; * *p* < 0.05, ** *p* < 0.01, *** *p* < 0.001.

The morphology of the hippocampus and SNpc was studied via H&E staining (Figure 2A). In control mice, hippocampal neurons were densely packed, regularly arranged, and showed intact spherical somata with distinct nuclear membranes. In contrast, MPTP-treated mice showed pathological changes, including disordered neuronal arrangement, uneven cell sizes, pyknotic nuclei, and blurred nuclear boundaries in both the hippocampus and the SNpc. Oral supplementation with AGP improved these structural abnormalities induced by MPTP, restored the compact and ordered neuronal sequence of the hippocampus, and showed more compact and ordered lamination. AGP-treated groups exhibited improved nuclear clarity and better cell morphology. These qualitative observations suggest that AGP diet effectively reduced MPTP-induced histological damage to neurons in key brain regions associated with PD.

The survival of dopaminergic neurons and the aggregation of pathological proteins were evaluated by immunofluorescence staining of TH and α-Syn (Figure 2A,B). Compared with control mice, the relative IOD of the TH-positive staining significantly decreased (*p* < 0.01), while the α-Syn aggregation was significantly increased (*p* < 0.001) in MPTP-treated mice, indicating severe dopaminergic neurodegeneration and pathological protein deposition. Notably, AGP supplementation effectively reversed these pathological alterations. Compared to the MPTP model group, the IOD of the TH-positive staining was significantly restored in the AGPL (*p* < 0.01) and AGPM (*p* < 0.01) groups. Concurrently, α-Syn aggregation was significantly suppressed in the AGPL (*p* < 0.001) and AGPM (*p* < 0.001) groups. These results suggest that in MPTP-induced PD mice, AGP dietary supplementation has strong neuroprotective benefits by preserving dopaminergic neuron integrity and preventing aberrant α-Syn accumulation.

The levels of DA and its metabolites DOPAC and HVA were measured in mouse brain tissue by ELISA (Figure 2C). MPTP-treated mice showed significant decreases in the levels of DA (*p* < 0.01), DOPAC (*p* < 0.001), and HVA (*p* < 0.001) compared with control mice, indicating severe dysfunction of the dopaminergic neurotransmitter system. AGP supplementation effectively reversed this trend. DA levels were significantly increased in the AGPL (*p* < 0.05), AGPM (*p* < 0.05), and AGPH (*p* < 0.01) groups compared to the MPTP model group. Similarly, DOPAC levels were significantly restored in the AGPL (*p* < 0.01), AGPM (*p* < 0.05), and AGPH (*p* < 0.001) groups, while HVA levels showed robust recovery in all AGP-supplemented groups (AGPL and AGPM, *p* < 0.01; AGPH, *p* < 0.001). Together, these results show that oral AGP treatment effectively restore the function of the dopaminergic neurotransmitter system in MPTP-induced PD mice and further support its potential as a nutritional intervention for PD.

### 3.3. AGP Supplementation Alleviates Oxidative Stress in PD Mouse Models

**Figure 3:**
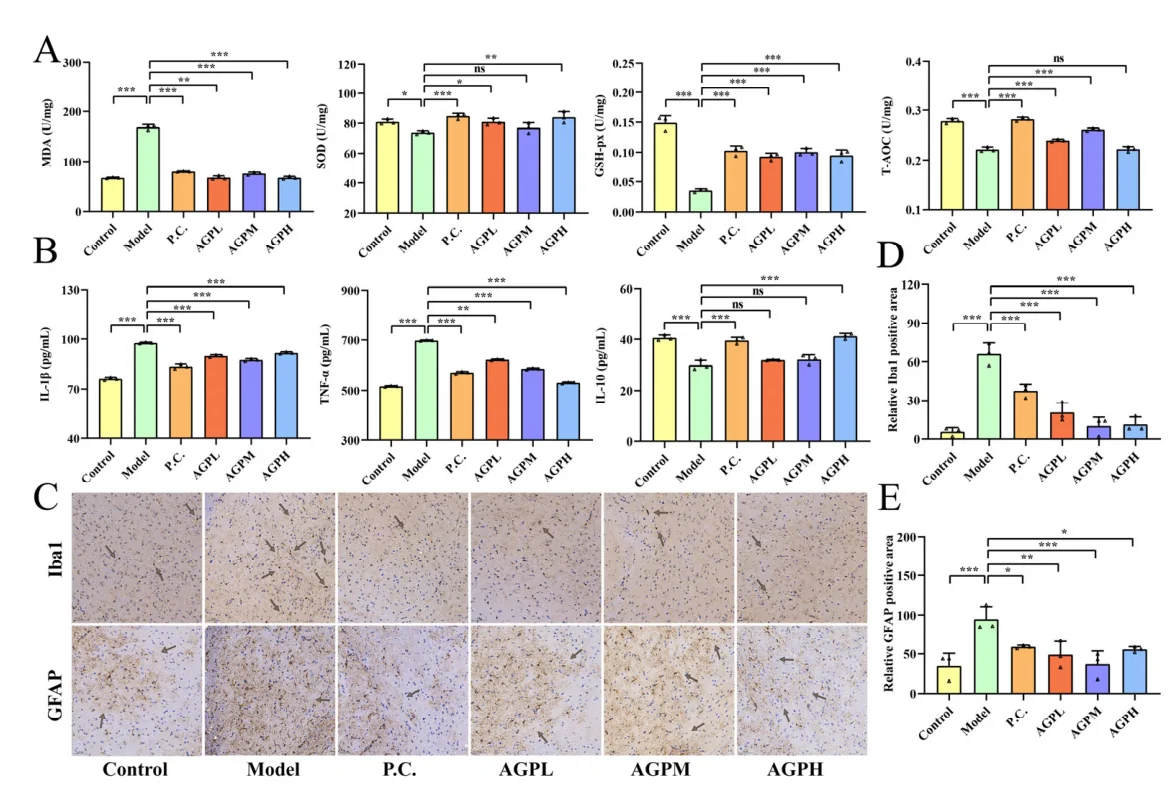
Dietary AGP supplementation alleviates oxidative stress and neuroinflammation in MPTP-induced PD mice. (**A**) Brain oxidative stress markers (MDA, SOD, GSH-Px, T-AOC). (**B**) Serum inflammatory cytokines (IL-1β, TNF-α, IL-10). (**C**) Iba1 and GFAP immunohistochemistry in the SNpc. (**D**) Iba1-positive cell counts. (**E**) GFAP-positive cell counts. Data are presented as mean ± SD (*n* = 3 per group for oxidative stress markers, inflammatory cytokine, and immunohistochemical analyses). ns, not significant; * *p* < 0.05, ** *p* < 0.01, *** *p* < 0.001.

To evaluate the regulatory effects of AGP dietary supplementation on oxidative stress in PD model mice, we measured biological markers related to oxidative stress in brain tissues (Figure 3A). Compared to control mice, the activities of SOD (*p* < 0.05), GSH-Px (*p* < 0.001), and T-AOC (*p* < 0.001) decreased significantly in PD model mice. Conversely, the level of MDA, a lipid peroxidation product, increased drastically (*p* < 0.001). These results indicate that MPTP causes severe oxidative imbalance in the brain and leads to increased oxidative damage. In particular, oral AGP supplementation effectively reversed these abnormalities of oxidative stress and systematically improved antioxidant defense capacity in PD model mice. Specifically, the AGPL (*p* < 0.05) and AGPH (*p* < 0.01) groups showed a considerable restoration of SOD activity. All groups supplied with AGP had significantly higher GSH-Px activity (*p* < 0.001), while AGPL and AGPM groups also had significantly higher T-AOC levels (*p* < 0.001). At the same time, MDA levels were significantly lower in the AGPL group (*p* < 0.01) and in the AGPM and AGPH groups (*p* < 0.001). Collectively, these findings show that dietary AGP reduces oxidative damage in the brains of MPTP-induced PD model mice by increasing the endogenous activity of antioxidant enzymes, enhancing total antioxidant capacity, and suppressing lipid peroxidation. This regulatory effect on oxidative stress may contribute to the potential neuroprotective effects of AGP as a dietary supplement for PD.

### 3.4. AGP Supplementation Mitigates Neuroinflammation in PD Mouse Models

To fully assess the anti-inflammatory effects of AGP dietary supplementation, we investigated both peripheral inflammatory factors and central glial cell activation. Figure 3B,C show a systemic imbalance in the inflammatory network: MPTP-induced PD model mice had significantly higher levels of pro-inflammatory cytokines (IL-1β and TNF-α) and a significant reduction in the anti-inflammatory cytokine IL-10 compared to the Control group. In particular, after supplementation with AGP, serum levels of TNF-α and IL-1β in PD mice decreased, while serum levels of IL-10 rose dramatically. These findings indicate that diet AGP suppresses the systemic inflammatory response by rebalancing pro-inflammatory and anti-inflammatory cytokines in the periphery. To determine whether the peripheral inflammation modulation was accompanied by the central glial responses, we performed immunohistochemical staining for Iba1 (a microglial marker) and GFAP (an astrocyte marker) in the SNpc (Figure 3C–E). The mice treated with MPTP exhibited a significant increase in Iba1-positive cells, accompanied by characteristic morphological changes in activated microglia, including expanded cell bodies and retracted processes. AGP supplementation significantly reduced microglial activation in the SNpc. Simultaneously, the MPTP model group exhibited much higher GFAP expression, an indicator of astrocyte reactivity, whereas AGP-treated mice showed much lower GFAP immunoreactivity than the model group. All of these results indicate that AGP, when taken as a dietary supplement, inhibits excessive activation of astrocytes and microglia, preventing cascade amplification of neuroinflammation in the central nervous system. This double control of the peripheral and central inflammatory response further supports the promise of AGP as a dietary intervention for PD.

### 3.5. AGP Supplementation Modulates Serum Metabolome in PD Model Mice

**Figure 4:**
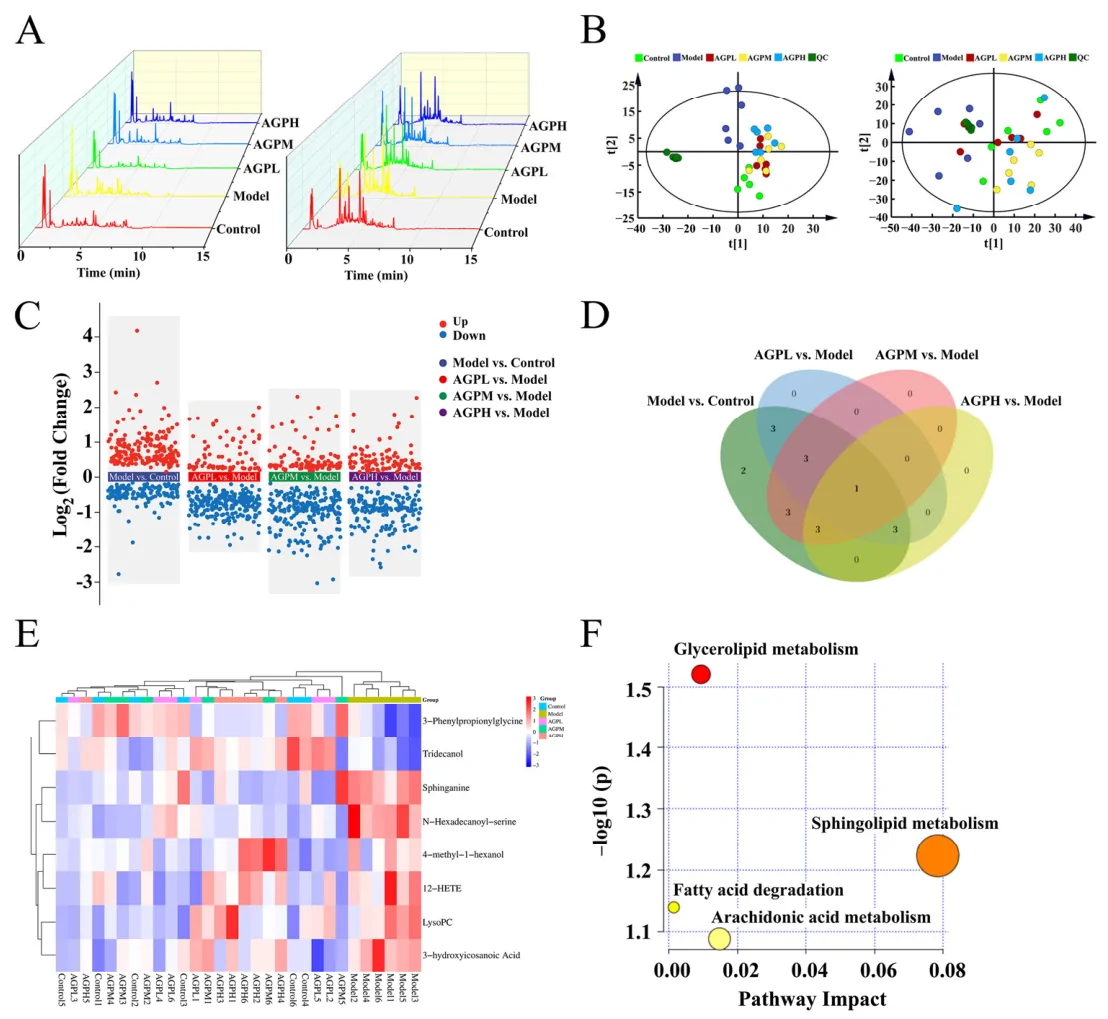
Dietary AGP supplementation restores serum metabolic profiles in MPTP-induced PD mice. (**A**) Representative TICs in positive and negative ion modes. (**B**) PCA score plots. (**C**) Volcano plot of differential metabolites (Model vs. Control). (**D**) Venn diagram of shared differential metabolites. (**E**) Heatmap of 8 key differential metabolites. (**F**) Pathway enrichment analysis (sphingolipid, glycerophospholipid, fatty acid, and arachidonic acid metabolism). Data are presented as mean ± SD (*n* = 6 per group for serum metabolomics analyses).

To investigate the systemic metabolic effects of dietary AGP supplementation, serum samples were subjected to non-targeted analysis. The total ion chromatogram (TIC) showed effective chromatographic separation of serum metabolites in all experimental groups, characterized by clear, well-resolved peaks and low, stable baselines, indicating exceptional data reproducibility and suitability for downstream analysis (Figure 4A and Figure S6A). To assess data replication, pooled quality control (QC) samples were injected periodically throughout the analysis process. The relative standard deviations (RSDs) of feature intensities in the QC samples were calculated (Figure S6C), and most features exhibited RSD values < 30%, indicating sufficient analytical reproducibility and suitability for subsequent analysis.

In addition, multivariate statistical analysis revealed different metabolic profiles between the experimental groups. PCA clearly distinguished between the control group and the PD model group, suggesting profound disturbances of serum metabolism in MPTP-induced mice. In particular, the AGP-supplemented groups clustered closely with the control group, indicating that dietary AGP effectively ameliorated MPTP-induced metabolic disturbances (Figure 4B).

To improve group discrimination and identify key discriminatory metabolites, orthogonal partial least squares discriminant analysis (OPLS-DA) was conducted. The OPLS-DA score plot exhibited robust separation between the MPTP group and both the control and AGP groups (Figure S4A,B). Two hundred permutation tests were conducted to validate the OPLS-DA models. In positive ion mode, the R^2^ intercepts for MPTP vs. Control, AGPL vs. MPTP, AGPM vs. MPTP, and AGPH vs. MPTP were 0.596, 0.617, 0.612, and 0.578, respectively, and the corresponding Q^2^ intercepts were −0.681, −0.635, −0.676, and −0.672; in negative ion mode, the R^2^ intercepts were 0.555, 0.614, 0.538, and 0.658, and the Q^2^ intercepts were −0.55, −0.635, −0.69, and −0.442, demonstrating robust model performance without overfitting (Figure S4C). Volcano plots showed the distribution of significantly altered serum metabolites, with blue and red dots representing downregulated and upregulated species, respectively (Figure 4C,D). Variable importance in projection (VIP) > 1.0, Benjamini–Hochberg FDR *<* 0.15, and |FC| > 1.2 were used to filter potential biomarkers. The Venn diagram showed that 16 differential metabolites were shared between the MPTP vs. Control and AGP vs. MPTP comparisons. Among these 16 common metabolites, 8 were identified by MS/MS spectral matching, indicating that they were potential key serum biomarkers of MPTP-induced PD (Figure 4D and Tables S7–S11). The hierarchical clustering heatmap showed the expression patterns of these 8 key metabolites, revealing that in comparison to the Control group, six were significantly upregulated and two downregulated in the PD model group. Importantly, AGP supplementation reversed the abnormal levels of 8 out of these 8 metabolites and strongly supported its neuroprotective role by modulating serum metabolic homeostasis (Figure 4E). These differential metabolites were mainly concentrated in four important metabolic pathways: sphingolipid metabolism, glycerophospholipid metabolism, fatty acid degradation, and arachidonic acid metabolism, according to pathway enrichment analysis using MetaboAnalyst 6.0 (Figure 4F). It is known that the dysregulation of these pathways reflects impaired membrane integrity, oxidative stress, and neuroinflammatory processes in PD, while their restoration by AGP supplement suggests an association with its neuroprotective potential as a dietary supplement.

### 3.6. AGP Supplementation Reshapes Fecal Metabolic Profiles in MPTP-Induced PD Mice

**Figure 5:**
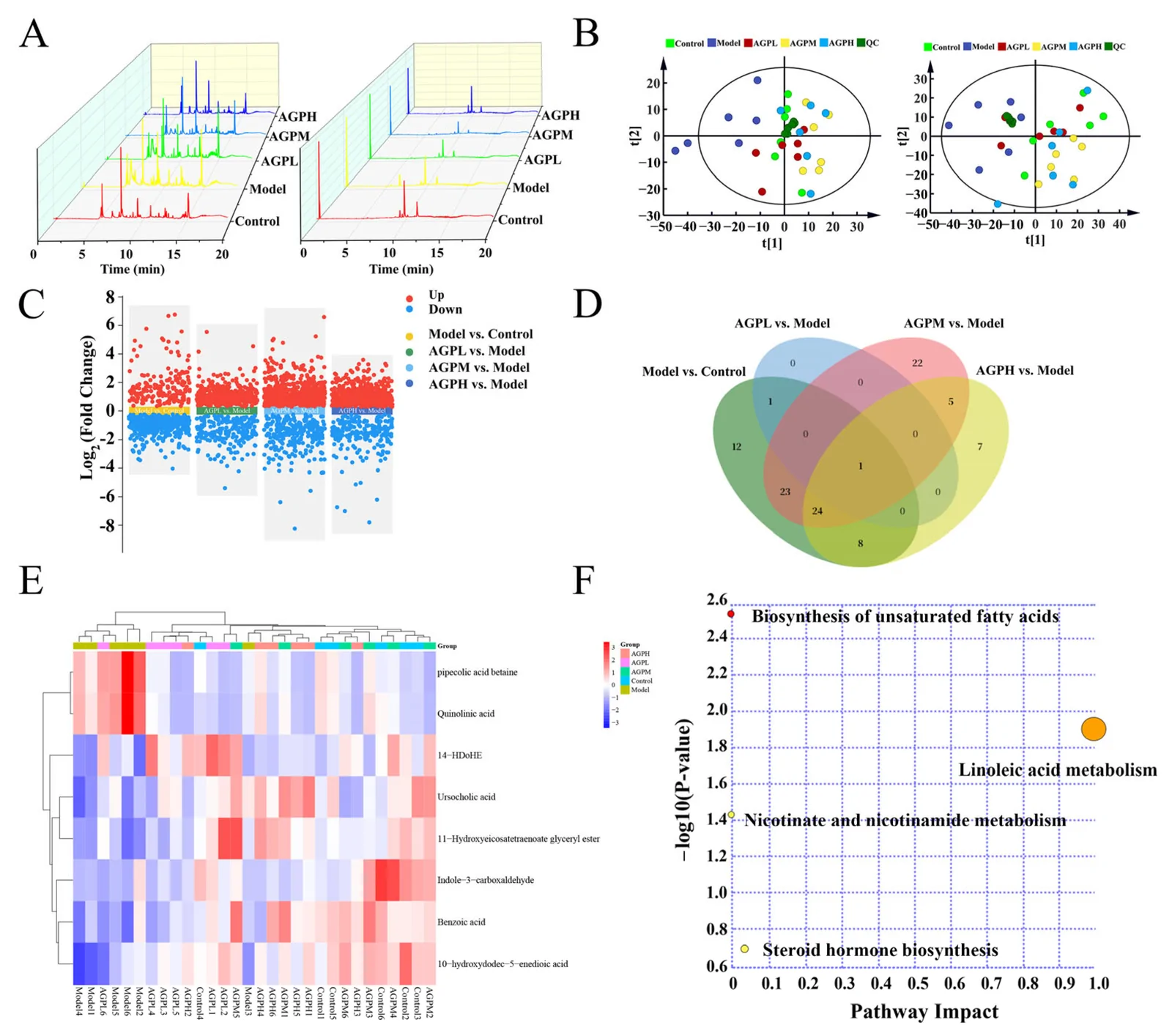
Dietary AGP supplementation restores fecal metabolic profiles in MPTP-induced PD mice. (**A**) Representative TICs in positive and negative ion modes. (**B**) PCA score plots. (**C**) Volcano plot of differential metabolites (Model vs. Control). (**D**) Venn diagram of shared differential metabolites. (**E**) Heatmap of 8 key differential metabolites. (**F**) Pathway enrichment analysis (biosynthesis of unsaturated fatty acids, linoleic acid metabolism, nicotinate and nicotinamide metabolism, steroid hormone biosynthesis). Data are presented as mean ± SD (*n* = 6 per group for fecal metabolomics analyses).

To dissect the intestinal metabolic disturbances underlying MPTP-induced PD and the modulatory effects of dietary AGP supplementation, we conducted nontargeted metabolic profiling of fecal samples. Multivariate statistical analyses, including PCA and OPLS-DA, revealed significant metabolic differences between the Control group and MPTP model (Figure 5 and Figures S5A,B and S6B,D). In particular, the AGP-supplemented groups shifted toward Control group in PCA score plots, indicating that dietary AGP effectively reverses MPTP-induced metabolic dyshomeostasis by remodeling the intestinal metabolic network. To verify the OPLS-DA models, 200 permutations were conducted (Figure S5C,D). In positive ion mode, the R^2^ intercepts for MPTP vs. Control, AGPL vs. MPTP, AGPM vs. MPTP, and AGPH vs. MPTP were 0.835, 0.829, 0.714, and 0.77, respectively, and the corresponding Q^2^ intercepts were −0.421, −0.399, −0.505, and −0.367. In negative ion mode, the R^2^ intercepts were 0.8, 0.758, 0.732, and 0.792, and the Q^2^ intercepts were −0.222, −0.34, −0.37, and−0.343. These results show robust model performance for most comparisons, as indicated by the negative Q^2^ intercepts and R^2^ intercepts being substantially lower than the corresponding R^2^Y values. These permutation results confirm that the OPLS-DA models were not overfitted.

Using the screening criteria (VIP > 1.0, Benjamini–Hochberg FDR < 0.25, |FC| > 1.2), the analysis of the Volcano plots and Venn diagram showed that there were 57 differential fecal metabolites overlapped between the comparisons of MPTP vs. Control and AGP vs. MPTP, 8 of which were further identified by MS/MS spectral matching (Figure 5C,D and Tables S12–S16). According to a hierarchical heatmap, MPTP model groups showed 2 upregulated and 6 downregulated metabolites in comparison to the control group. Importantly, AGP supplementation significantly reversed the abnormal levels of most of these metabolites (Figure 5E).

These differential metabolites were mainly involved in important metabolic pathways, such as biosynthesis of unsaturated fatty acids, linoleic acid metabolism, nicotinate and nicotinamide metabolism, and steroid hormone biosynthesis according to pathway enrichment analysis (Figure 5F). Since these pathways are closely linked to gut microbial metabolism and host–microbe crosstalk, the modulation of these pathways by AGP supplementation suggests that dietary AGP exerts systemic metabolic benefits by restoring intestinal homeostasis, further supporting its potential as a nutritional intervention for PD.

### 3.7. AGP Supplementation Reverses MPTP-Induced Gut Microbiota Dysbiosis in PD Model Mice

**Figure 6:**
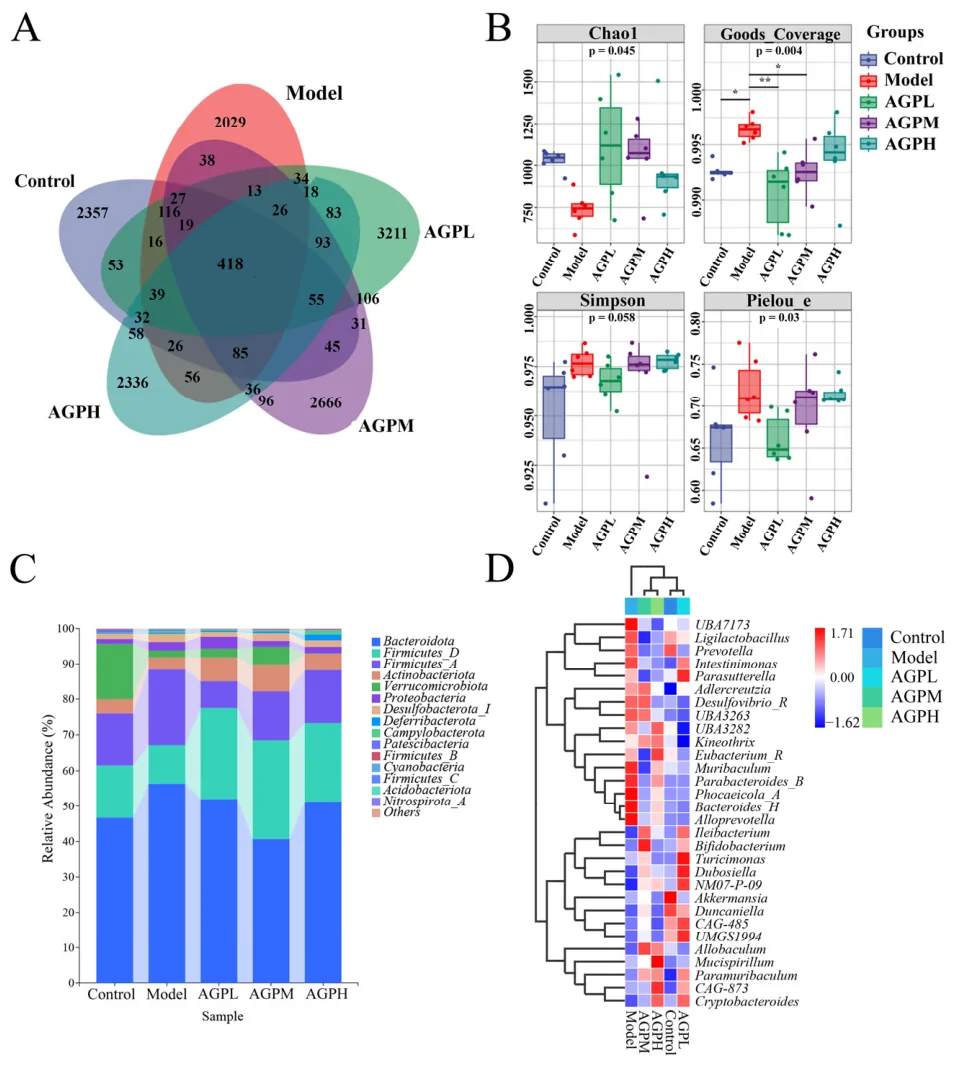
Dietary AGP supplementation modulates gut microbiota diversity and composition in MPTP-induced PD mice. (**A**) Venn diagram of shared and unique ASV. (**B**) Alpha diversity indices (Chao1, Good’s coverage, Simpson, Pielou’s evenness). (**C**) Phylum-level relative abundance. (**D**) Genus-level heatmap of microbial taxa. Data are presented as mean ± SD (*n* = 6 per group for gut microbiome analyses). * *p* < 0.05, ** *p* < 0.01.

To investigate whether the neuroprotective effect of dietary AGP supplementation is associated with modulating gut microbial community composition, 16S rRNA gene sequencing was utilized on fecal samples from experimental mice. Rarefaction curves indicated that the sequencing depth was sufficient for downstream analyses (Figure S7A). ASV analysis showed that MPTP exposure significantly reduced intestinal microbial diversity, whereas AGP supplementation restored the number of ASV to varying degrees, suggesting the critical role of dietary AGP in preserving gut microbial community abundance in PD mice (Figure 6A). Beta diversity was evaluated using PCoA and NMDS based on Bray–Curtis distance matrices (Figure S7C,D). PERMANOVA (Figure S7B and Table S6) confirmed significant differences in the structure of the microbial community between groups (*p* < 0.05), and both ordination methods revealed distinct clustering of the Control group and MPTP model group, indicating that MPTP treatment induced profound structural changes in the gut microbiota. In contrast, all AGP-supplemented groups (AGPL, AGPM, AGPH) showed a clear tendency to shift toward the Control group, indicating that dietary AGP partially mitigated MPTP-induced gut microbial dysbiosis and promoted the community toward a normal state. Alpha diversity analysis (Figure 6B) further revealed significant changes in the Chao1 index, Good’s coverage index, and Pielou’s evenness index between MPTP model mice and control mice. In particular, AGP supplementation restored these indicators to levels close to those of the Control group. Together, our results show that dietary AGP can reverse intestinal microbiota imbalance caused by MPTP and promote the restoration of a normal community structure.

To gain deeper an understanding of gut microbial community composition, the phylum-level taxonomic structure of the intestinal microbiota was examined (Figure 6C). The two most common phyla, *Bacteroidetes* and *Firmicutes*, accounted for about 76% of the total bacterial community, while *Actinobacteria*, *Spirochaetes*, and *Proteobacteria* represented other key phyla. MPTP exposure induced profound shifts in the relative abundances of these phyla compared with the control, whereas AGP supplementation effectively reversed this dysbiosis and restored phylum-level profiles to near-control levels. Furthermore, cluster analysis at the genus level and heatmap visualization were employed to characterize specific microbial changes in MPTP-induced PD mice (Figure 6D). The MPTP group formed a distinct cluster that was significantly separated from the Control group.

To further characterize the differences in intestinal microbial communities among the Control group, Model group, and AGP-supplemented groups, we conducted linear discriminant analysis effect size (LEfSe) analysis (Figure S8A). In contrast to the Control group, the Model group showed significantly higher levels of *Christensenella*, unclassified *Oscillospiraceae*, *Mailhella*, unclassified *Enterobacteriaceae_A*, *Bacteroides_H*, unclassified *Muribaculaceae*, unclassified *Bacteroidaceae*, *Anaerofustis, Holdemania*, and *Lachnoclostridium_B* (*p* < 0.05), whereas the abundance of *Allobaculum* was markedly decreased. This microbial taxonomic imbalance is closely associated with PD pathogenesis, with neuroinflammation emerging as a critical downstream consequence of this dysbiosis. Importantly, AGP supplementation led to a shift in genus-level community composition toward Control levels, with all AGP-supplemented groups clustering closely with the Control group and effectively reversing the dysregulation of these key taxa caused by MPTP. Taken together, these results suggest that dietary AGP effectively reverses MPTP-induced gut microbial structural disruption, further supporting its potential as a nutritional intervention targeting gut microbiota in PD.

### 3.8. Correlation Analysis Links Gut Microbial Taxa to Behavioral, Inflammatory, and Oxidative Stress Parameters

**Figure 7:**
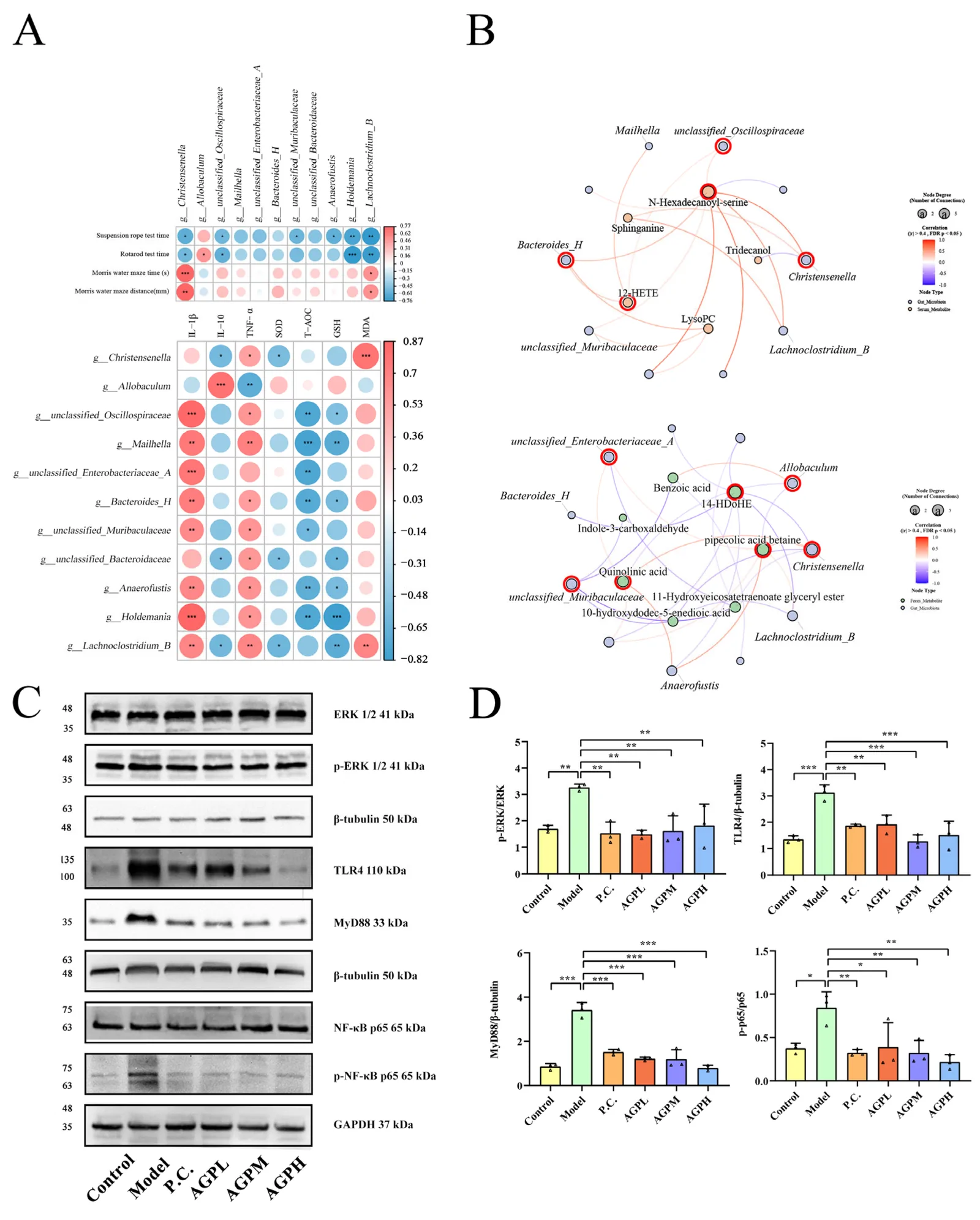
Dietary AGP supplementation modulates gut microbiota–host interactions and neuroinflammatory signaling in PD mice. (**A**) Spearman correlation heatmap of gut microbial genera with biochemical parameters. (**B**) Correlation network of gut microbiota and differential metabolites. (**C**) Representative Western blot bands and quantification of TLR4, MyD88, p-p65/p65, and p-ERK/ERK in the SNpc. The cropped membrane images with molecular weight markers and lane annotations are provided in Supplementary Figure S9). (**D**) Quantitative analysis of protein expression levels. Data are presented as mean ± SD (*n* = 6 per group for correlation analyses; *n* = 3 per group for Western blotting). * *p* < 0.05, ** *p* < 0.01, *** *p* < 0.001.

Spearman correlation analysis was used to determine the potential correlations between gut microbiota and behavioral dysfunction, as well as inflammatory and oxidative stress biomarkers in PD model mice. As shown in the heatmap (Figure 7A), significant correlations were found between specific gut microbial genera and multiple behavioral and biochemical indices. Interestingly, the relative abundance of *Christensenella* was positively associated with the pro-inflammatory cytokine TNF-α and the pro-oxidant marker MDA, but negatively associated with the antioxidant enzyme SOD and the anti-inflammatory cytokine IL-10. Furthermore, the abundance of *Christensenella* showed a strong positive association with motor deficits, as assessed by the suspension test and rotarod test. *Bacteroides_H* also showed strong negative association with T-AOC and GSH and significant positive association with the pro-inflammatory cytokines TNF-α and IL-1β. Additionally, *Lachnoclostridium_B* showed a negative correlation with IL-10, SOD, and GSH and a strong positive association with TNF-α, MDA, and escape latency in the Morris water maze test. Collectively, these association patterns indicate that in PD model mice, changes in the abundance of particular gut bacterial genera are strongly associated with oxidative stress, neuroinflammation levels, and behavioral abnormalities. Importantly, these results suggest that the neuroprotective effects of dietary AGP supplementation may be associated with its capacity to modify important intestinal microbial composition, thereby modulating downstream inflammatory signaling and cytokine release (including TNF-α and IL-1β), ultimately helping to alleviate behavioral and biochemical abnormalities resembling PD.

### 3.9. AGP Supplementation Modulates Microbiota–Metabolite Networks via the MGBA

To further explore associations within the MGBA axis in PD, we combined Spearman correlation analysis with a network of co-occurrence to integrate gut microbiome data with key metabolites from serum and feces (Figure 7B). Serum metabolite–microbiota network analysis revealed that several key bacterial species, including *Christensenella*, *Bacteroides_H*, and unclassified *Oscillospiraceae*, were strongly associated with differential serum metabolites such as putative N-hexanoylserine and putative 12-hydroxyeicosatetraenoic acid (12-HETE). Since neuroinflammation is a central pathological driver of PD, we further examined metabolites associated with inflammatory signaling. Notably, the pro-inflammatory eicosanoid derivative putative 12-HETE was significantly positively correlated with several bacterial species, including *Bacteroides_H*, *Mailhella*, and unclassified *Oscillospiraceae*. Furthermore, putative serine, a key substrate in the sphingolipid de novo synthesis pathway that can promote the production of pro-inflammatory ceramides, was positively associated with unclassified *Muribaculaceae, Lachnoclostridium_B* and *Christensenella*. Fecal metabolite–microbiota network analysis identified 14-HDoHE, pipecolic acid betaine, and quinolinic acid as the most highly connected fecal metabolites, while *Christensenella* and unclassified_*Muribaculaceae* were the most highly connected genera. 14-HDoHE was negatively correlated with seven genera, including *Bacteroides_H*, *Lachnoclostridium_B*, and *Christensenella*, whereas pipecolic acid betaine and quinolinic acid were positively correlated with *Christensenella*, *Anaerofustis*, and unclassified_E*nterobacteriaceae_A*, and negatively correlated with *Allobaculum*. Collectively, these network analyses suggest that AGP supplementation may be associated with alterations in important gut microbial taxa and their related pro-inflammatory or neuroactive metabolites via MGBA-related pathways, which may contribute to its neuroprotective effects. However, causality cannot be established from the present data, and further studies are needed to verify the potential of AGP as a nutrition intervention in PD to target host–microbe metabolic crosstalk.

### 3.10. AGP Supplementation Is Associated with Reduced TLR4/MyD88/NF-κB and ERK Signaling Activation in MPTP-Induced PD Mice

To further examine inflammatory signaling changes associated with dietary AGP supplementation, we explored key inflammatory pathways in the SNpc by Western blotting. As shown in Figure 7C,D, the TLR4/MyD88/NF-κB inflammatory signaling cascade was strongly activated in the SNpc of MPTP-induced mice, the expression of TLR4 and MyD88 and the ratio of p-p65/p65 were increased, and the phosphorylation of ERK (p-ERK/ERK) was abnormally elevated. In particular, oral AGP supplementation effectively reversed these pathological signaling perturbations and restored pathway activity to Control levels. Collectively, these findings suggest possible associations between gut microbial ecology and central neuroinflammatory signaling. Specifically, the AGP supplement selectively modulates key gut microbial taxa, including *Christensenella*, while simultaneously reducing the levels of pro-inflammatory metabolites such as putative 12-HETE and putative serine. This regulation may prevent excessive activation of TLR4/MyD88/NF-κB pathways and ERK phosphorylation, potentially blocking the transmission of peripheral inflammatory signals to the brain and thereby potentially contributing to neuroprotective effects in PD-model mice.

## 4. Discussion

The structural characteristics of AGP (Figure S2) provide compositional information relevant to its potential neuroprotective effects as a dietary bioactive protein. AGP is composed of 39.39% hydrophobic amino acids, which are linked to anti-inflammatory and neuroprotective effects [25]. Notably, AGP contains relatively high levels of aspartic acid (Asp) and leucine (Leu), which are associated with CNS protection and nutrient regulation, respectively [26,27]. From a nutritional perspective, these amino acid profiles suggest that AGP, when administered as an oral dietary supplement, may act as a source of precursors for the production of bioactive SCFAs and other neuroactive metabolites by gut microbes. Deconvolution of the CD spectra (Table S4) revealed that β-sheets constituted 56.6% of the secondary structure, with α-helices (14.7%), random coils (9.3%), and turns (19.4%) accounting for the remainder. This conformational feature may contribute to enhanced resistance to gastrointestinal proteases, although this remains a hypothesis as proteolytic resistance was not directly measured [28]. Furthermore, two-dimensional electrophoresis identified six core functional proteins in AGP (Table S3). Among these proteins, phosphoglycerate kinase 1 (PGK1), a key glycolytic enzyme, supports ATP generation in neurons. PGK1 is upregulated and regulated by the PD-related molecular chaperone PARK7 (DJ-1), and its upregulation has been shown to alleviate synaptic dysfunction in PD models [29,30]. Another glycolytic enzyme, GAPDH, also has neuroprotective potential and can influence apoptosis and responses to oxidative stress [31].

On the basis of the structural characteristics outlined above, this study utilized an MPTP-induced PD mouse model to evaluate the ameliorative effects of oral AGP supplementation on both motor dysfunction and neuropathological features. MPTP treatment induced motor coordination disorders, impaired balance, and impaired place navigation performance in mice. These behavioral abnormalities partially resemble the common motor retardation and postural instability observed in PD patients, thereby providing behavioral validation for the effectiveness of the model. In this model, dopaminergic neurons in the SNpc were damaged, accompanied by the pathological accumulation of α-Syn, leading to reduced dopamine synthesis and neural transmission disorders [32]. Our data indicate that AGP dietary intervention was associated with a reversal of behavioral defects in PD mice and improvement in core pathological features. Specifically, AGP significantly reduced neuronal loss and structural disorganization in the SNpc and hippocampus while preserving TH-positive neurons. These results are consistent with a major pathological feature of dopaminergic neuron loss in Parkinson’s disease. TH, as the rate-limiting enzyme for dopamine synthesis, plays a crucial role, and its continuous reduction directly leads to motor regulation disorders [33]. Additionally, AGP was associated with reduced the abnormal aggregation of α-Syn and with changes consistent with attenuation of the neurotoxic cascade, thereby providing dual protection for dopaminergic neurons. AGP treatment also increased the levels of dopamine and its metabolites, directly ameliorating motor deficits. Reduced dopamine synthesis is a core cause of motor symptoms in PD [34]. AGP was associated with preserving TH-positive neurons and reduced α-Syn aggregation. Furthermore, AGP partially reversed the place navigation deficits induced by MPTP, suggesting potential benefits for hippocampal neuron protection. However, these results should be interpreted cautiously, as the protocol used visible platform training prior to the submerged platform test and did not include a probe trial, which limits conclusions about spatial memory retention. Collectively, these findings indicate that AGP, as a dietary supplement, was associated with improvements in motor function and place navigation performance.

Oxidative stress is a key mechanism contributing to the onset and progression of PD. One of the main pathogenic characteristics of PD is the death of dopaminergic neurons in the SNpc caused by excessive activation of oxidative stress. The body’s systemic capacity to reduce oxidative damage and neutralize free radicals is reflected in T-AOC [35,36]. The brain contains high concentrations of easily oxidizable polyunsaturated fatty acids, and the by-product of lipid peroxidation, MDA, can damage mitochondrial function. MDA serves as an important marker of oxidative damage [37]. ROS play a role in signalling under normal physiological conditions. However, excessive production of ROS under pathological conditions can disrupt the redox balance of cells, leading to neuronal loss and increased proliferation of astrocytes [37]. GSH-Px helps protect cell membranes from oxidative damage. An imbalance in oxidative stress is indicated by elevated MDA levels and decreased SOD, GSH-Px, and T-AOC activity in PD animal models [38,39]. In our study, MPTP-induced PD model mice recapitulated this pathological signature, with corresponding decreases in SOD, GSH-Px, and T-AOC activity and markedly elevated brain MDA levels, confirming the presence of robust oxidative stress in this model. After AGP intervention, these indicators showed significantly improvement, suggesting that AGP may be associated with neuroprotection, accompanied by reducing lipid peroxidation and enhancing endogenous antioxidant defenses. Given that dietary antioxidants derived from natural plant proteins have attracted considerable interest in nutritional neuroscience, our results position AGP as a potential plant-derived ingredient for combating oxidative damage in neurodegenerative conditions.

Neuroinflammation is a central factor in the pathology of PD, and is characterized by the aberrant activation of astrocytes and microglia and increased levels of pro-inflammatory cytokines such as TNF-α and IL-1β. Microglia, the main immune cells of the central nervous system, are essential for preserving neural homeostasis [40,41,42]. Numerous studies have shown that increased proliferation of reactive microglia, increased expression of pro-inflammatory markers, and increased infiltration of macrophages in the SNpc are common in PD patients [43]. In our study, AGP intervention significantly reversed these inflammatory responses, as evidenced by decreased levels of pro-inflammatory cytokines (IL-1β and TNF-α) and increased production of the anti-inflammatory factor IL-10. These changes were accompanied by lower Iba1 and GFAP immunoreactivity, suggesting reduced microglial and astrocytic activation. Given that peripheral inflammatory signals can be transmitted to the CNS and influence the activation of immune cells, this study further investigated the activation status of microglia and astrocytes in the SNpc, which is the main lesion area affected in PD. Immunostaining results demonstrated that AGP significantly reduced the abnormal activation of Iba1-positive microglia and GFAP-positive astrocytes in the SNpc, suggesting that AGP can suppress the amplification of the CNS inflammatory cascade. These results highlight the potential of AGP as an immunomodulatory dietary component that may temper neuroinflammation through the coordinated regulation of glial cell phenotypes.

Before the onset of typical motor symptoms, PD patients often experience a prolonged history of gastrointestinal dysfunction, suggesting a potential role of gut microbiota dysbiosis in disease progression through the MGBA [44,45]. This connection provides a compelling rationale for the use of orally administered nutritional supplements such as AGP to modulate the gut ecosystem and indirectly influence brain health. In an MPTP-induced PD mouse model, significant dysbiosis of the intestinal microbiota and systemic metabolic disorders were detected. Research has demonstrated that the intestinal microbiota of PD patients is significantly altered, with increases in the abundance of pro-inflammatory genera such as *Enterobacteriaceae* and *Christensenella* and a decrease in the abundance of probiotic genera such as *Allobaculum* [46]. This microbiota imbalance is closely linked to impaired intestinal barrier function and neuroinflammation, including microglial cell activation and astrocyte proliferation [47]. In MPTP-induced mice, we found that the intestinal microbial proliferation richness and diversity were significantly reduced, and that the abundance of particular species changed noticeably. Notably, the abundance of *Christensenella* and unclassified *Enterobacteriaceae_A* significantly increased, whereas that of *Allobaculum* sharply decreased. The abundance of *Christensenella*, a Gram-negative anaerobic bacterium, was increased in the fecal microbiota of clinical PD patients [47]. Its excessive proliferation may disrupt intestinal anaerobic conditions, promoting the overgrowth of facultative anaerobes (e.g., *Enterobacteriaceae*). This can trigger local inflammation, activate intestinal immune cells, and promote the release of pro-inflammatory substances (e.g., TNF-α) [47,48,49]. On the other hand, unclassified *Enterobacteriaceae_A*, owing to its ability to produce nitrite, may facilitate the aggregation of α-Syn in intestinal epithelial cells, providing a possible mechanistic link between microbiota imbalance and PD core pathology [50]. Concurrently, the abundance of *Allobaculum*, which possesses potential probiotic properties, significantly decreased. This reduction may be related to impaired intestinal barrier function [51,52]. Notably, oral AGP supplementation effectively reversed these microbiota alterations, restoring diversity and balance. These findings suggest that modulation of the gut microbiota may be associated with the neuroprotective effects of AGP, providing a basis for further investigation of “nutritional microbiota rewiring” as a potential strategy for PD management.

Non-targeted metabolomics analysis revealed that AGP intervention was associated with regulatory effects on key metabolic pathways, including the neuroinflammatory axis, metabolites, and gut microbiota in the PD model. Specifically, MPTP-induced PD model mice had considerably increased serum levels of the pro-inflammatory mediator putative 12-HETE. This metabolite exacerbated neuroinflammatory responses by activating the ILK/NF-κB pathway [53], and the prevalence of pro-inflammatory bacterial taxa, including *g__Mailhella*, was positively correlated with its levels. This correlation suggests that the gut microbiota may shift towards a pro-inflammatory state by regulating arachidonic acid (AA) metabolism. Conversely, the level of putative 14-HDoHE, which possesses anti-inflammatory properties, decreased in feces and was negatively correlated with bacteria such as *Bacteroides_H, Lachnoclostridium_B, Christensenella,* unclassified_*Oscillospiraceae,* unclassified*_Bacteroidaceae, Anaerofustis and Mailhella.* AGP intervention reversed the abnormal levels of these metabolites, suggesting its potential to regulate the pro-inflammatory/anti-inflammatory microenvironment by reshaping the metabolic balance of AA. Additionally, the level of putative sphinganine in the serum of PD mice was significantly increased. As a ceramide precursor, its accumulation indicated a disturbance in sphingolipid metabolism that may contribute to mitochondrial-dependent apoptosis and NF-κB-mediated inflammatory cascades [54], and these changes were positively correlated with the abundance of *Lachnoclostridium_B* and *Christensenella*. AGP was able to reduce putative sphinganine levels, suggesting that abnormal sphingolipid metabolism potentially inhibited the neuronal apoptosis signalling pathway. Moreover, correlation network analysis showed that 14-HDoHE was negatively correlated with *Bacteroides_H*, *Lachnoclostridium_B*, and *Christensenella*. Because 14-HDoHE is an anti-inflammatory lipid mediator, these negative associations suggest that AGP supplementation may be accompanied by changes in specific gut taxa related to a less pro-inflammatory metabolic environment. However, these findings are correlative, and causality remains to be established. From a nutritional standpoint, these metabolomic shifts suggest that AGP may act as a systemically active food-derived regulator that contributes to host–microbe metabolic homeostasis, rather than merely as a single-target compound [55].

The interaction between intestinal microbiota dysbiosis and systemic metabolic disturbances may be associated with neuroinflammation. Western blotting analysis of SNpc tissue revealed that, in MPTP-induced PD model mice, TLR4 and MyD88 expression and the p-p65/p65 ratio were increased, and ERK phosphorylation was elevated. In addition, serum levels of IL-1β and TNF-α were increased, as measured by ELISA. These findings are consistent with an association among intestinal-derived signals, peripheral inflammation, and central neuroinflammatory signaling. Our findings suggest that AGP may be associated with both peripheral and central changes. At the peripheral level, AGP was associated with a relative reduction in pro-inflammatory bacteria, such as those in the *Christensenella* genus, while the abundance of beneficial bacteria was restored. These changes were associated with modulation of the abnormal metabolism of AA, sphingolipids, phenylalanine, and bile acids, along with reduced levels of pro-inflammatory metabolites and neurotoxic substances. These changes were associated with lower TLR4/NF-κB pathway in SNpc tissue, which may potentially attenuate the amplification of neuroinflammatory responses. Crucially, this dietary intervention strategy may avoid the need for direct delivery of large protein molecules across the blood–brain barrier and may offer a complementary strategy to single-target central pharmacological treatments by targeting the systemic gut–brain axis. The potential role of AGP as a dietary supplement for integrative nutritional intervention in PD is supported by these findings, although causality remains to be established. However, it is crucial to note that this study has several limitations. First, the multi-omics findings are associative, not causal. The proposed MGBA-related interpretation remains a hypothesis. Fecal metabolomics was exploratory with a permissive Benjamini–Hochberg FDR threshold (q < 0.25), so these findings are hypothesis-generating and pathway-level conclusions should be interpreted cautiously. Second, severe MPTP toxicity led to death or euthanasia in 12 of the 60 mice. The systemic toxicity and selective survival may have influenced the behavioral, metabolic, microbiota, and inflammatory results. Survival bias cannot be fully excluded; results reflect surviving animals, and larger studies with survival analyses are needed in the future. In addition, the sample size for molecular analyses, particularly Western blotting with *n* = 3 per group, limits statistical power and represents a major limitation. Only male mice were used to avoid estrous-cycle confounding, and cage effects were not modeled. Future studies should include both sexes and account for cage effects. Third, AGP is a protein-enriched fraction, not a pure protein. Residual non-protein components may contribute to its effects, and the active constituents remain unknown. The preclinical doses used here exceed those from the recommended whole-root dose, possibly due to lower oral bioavailability of the purified fraction. They are not human recommended doses, and future trials should begin at lower doses with pharmacokinetic and safety monitoring. Fourth, in the Morris water maze, swimming velocity did not differ significantly among groups. However, motor impairment cannot be completely excluded as a contributor to escape latency and swimming distance, and no probe trial was included. These parameters were therefore interpreted as place navigation performance, not pure cognitive measures. Future research should therefore conduct well-designed human dietary intervention trials to assess the long-term safety, bioavailability, and effectiveness of AGP as a dietary supplement. Additionally, the integration of technologies such as fecal microbiota transplantation and metabolomic tracking would help identify key microbial targets and metabolic markers, thus potentially accelerating the advancement of precision medicine for PD.

## 5. Conclusions

In an MPTP-induced animal model of PD, this study suggests that dietary supplementation with AGP is associated with a variety of neuroprotective effects. AGP administration significantly improved motor function and place navigation performance, preserved dopaminergic neurons, reduced α-Syn aggregation, alleviated neuroinflammation and oxidative stress, and restored gut microbial homeostasis. In particular, AGP was associated with the regulation of key taxa, including *Christensenella, Mailhella*, and *Allobaculum*, which were closely linked to systemic metabolic homeostasis. Integrated multi-omics analyses revealed that AGP was associated with reshaping the gut microbiota and correcting host metabolic disorders, particularly in sphingolipid, arachidonic acid, phenylalanine, and bile acid pathways, which were associated with the suppression of central TLR4/MyD88/NF-κB and ERK signaling pathways. These results suggest that AGP may be associated with reduced pathogenesis of PD, with concurrent changes in MGBA-related pathways. In conclusion, AGP represents a promising, naturally derived candidate for further investigation as a dietary intervention for neurodegenerative diseases, but human trials are needed to evaluate its bioavailability, safety, and long-term efficacy.

## Data Availability

The untargeted metabolomics data have been deposited in MetaboLights under accession number MTBLS15946. The 16S rRNA gene sequencing data have been deposited in the NCBI Sequence Read Archive under BioProject accession number PRJNA1540443.

## Supplementary Materials

The following supporting information can be downloaded at: https://www.mdpi.com/article/10.3390/nu18203412/s1. Figure S1: Flowchart of the experimental design and sample allocation; Figure S2: Characterization of AGP structure (A) Amino Acid Composition. (B) UV absorption spectrum. (C) FTIR spectrum. (D) Intrinsic fluorescence spectrum. (E) CD spectrum (190-260 nm). (F) 2-DE electrophoresis map; Figure S3: Swimming speed in the Morris water maze test; Figure S4: Serum metabolite alterations in PD mice. (A) OPLS-DA score plots (Positive ion mode). (B) OPLS-DA score plots (Negative ion mode). (C) Permutation test plot (Positive ion mode, 200 Permutations). (D) Permutation test plot (Negative ion mode); Figure S5: Fecal metabolite alterations in PD mice (A) OPLS-DA score plots (Positive ion mode). (B) OPLS-DA score plots (Negative ion mode). (C) Permutation test plot (Positive ion mode, 200 Permutations). (D) Permutation test plot (Negative ion mode); Figure S6: PCA clustering and QC assessment of untargeted metabolomics data. (A,B) PCA plots of serum (A) and fecal (B) metabolomes derived from positive and negative ion modes. Separate 95% confidence ellipses were generated for each group (different from the global ellipse in the main text) to illustrate group-based sample clustering. (C,D) RSD distribution of metabolic features in QC samples. The x-axis presents RSD intervals (<10%, 10%–20%, 20%–30%, >30%), and the y-axis shows the number of corresponding features for serum (C) and feces (D); Figure S7: Sequencing saturation and bacterial community structural analysis. (A) Rarefaction curves reflecting sequencing depth and species saturation across samples. (B) PERMANOVA pairwise comparison results of microbial community dissimilarity. (C) PCoA plot based on Bray–Curtis distance matrices. (D) NMDS analysis; Figure S8: Linear discriminant analysis effect size (LEfSe) analysis and relative abundance of significantly differential bacterial genera among MPTP-induced PD mice, control group, and AGP treatment groups.; Figure S9: Original uncropped western blot membranes; Table S1: Preparation yield of AGP; Table S2. Mean body weights and working solution protein concentrations for AGP-treated groups during the treatment period. Table S3: Identification of six protein bands from AGP; Table S4: Secondary structure content of AGP at different pH values; Table S5: Statistics of sequence quality control and filtering procedures for 16S rRNA gene sequencing; Table S6: Pairwise PERMANOVA analysis of bacterial community structure based on Bray–Curtis distance; Table S7: Trends of differential metabolites in serum experimental groups; Table S8: Statistical parameters of differential metabolites in serum (Model vs. Control); Table S9: Statistical parameters of differential metabolites in serum (AGPL vs. Model); Table S10: Statistical parameters of differential metabolites in serum (AGPM vs. Model); Table S11: Statistical parameters of differential metabolites in serum (AGPH vs. Model); Table S12: Trends of differential metabolites in feces experimental groups; Table S13: Statistical parameters of differential metabolites in feces (Model vs. Control); Table S14: Statistical parameters of differential metabolites in feces (AGPL vs. Model); Table S15: Statistical parameters of differential metabolites in feces (AGPM vs. Model); Table S16: Statistical parameters of differential metabolites in feces (AGPH vs. Model).

## Abbreviations

The following abbreviations are used in this manuscript:

α-Syn	α-synuclein
AGP	American ginseng protein-enriched fraction
AA	arachidonic acid
CNS	central nervous system
DA	dopamine
DOPAC	dihydroxyphenylacetic acid
ERK	extracellular signal-regulated kinase
GAPDH	glyceraldehyde-3-phosphate dehydrogenase
MGBA	microbiota–gut–brain axis
GFAP	glial fibrillary acidic protein
GSH-Px	glutathione peroxidase
H&E	hematoxylin and eosin
HVA	homovanillic acid
IL-1β	interleukin-1β
IL-10	interleukin-10
MDA	malondialdehyde
MPTP	1-methyl-4-phenyl-1,2,3,6-tetrahydropyridine
MyD88	myeloid differentiation primary response 88
NF-κB	nuclear factor kappa B
OTUs	operational taxonomic units
P.C.	positive control
PCA	principal component analysis
PD	Parkinson’s disease
SCFAs	short-chain fatty acids
SNpc	substantia nigra pars compacta
SOD	superoxide dismutase
T-AOC	total antioxidant capacity
TH	tyrosine hydroxylase
TLR4	toll-like receptor 4
TNF-α	tumor necrosis factor α
VIP	variable importance in projection
