# Supplementary material for: American Ginseng Protein-Enriched Fraction Attenuates Neurobehavioral Deficits in MPTP-Induced Parkinson’s Disease Mice with Associated Modulation of Gut Microbiota and Metabolic Pathways
Source: Nutrients. 2026 Oct 18;18(20):3412. doi: 10.3390/nu18203412 (PMC13708459; doi:10.3390/nu18203412)
Supplement: Supplementary file 1 [file nutrients-18-03412-s001.zip › nutrients-4518090-supplementary.pdf]

# American ginseng protein Alleviates cognitive dysfunction on Parkinson's disease model mice via gut microbiota: brain-gut axis interactions

**Jie Li [1†], Xu Yang [1†], Jiahan Wang [1], Chunhui Yu [1], Jiahong Lu [1], Wenhui Lian [1*], Wei Liu[1*] and Yu Zhao [1*]**

1 Jilin Ginseng Academy, Changchun University of Chinese Medicine, Changchun, 130117, China

* Correspondence: lianwenhui1014@126.com (W. Lian), liuwei_cczyydx@163.com (W. Liu) and cnzhaoyu1972@126.com (Y. Zhao)

† These authors contributed equally to this work and should be considered co-first authors

## Supplementary

## Methods

**Preparation of American ginseng protein**

The dried American ginseng root (100 g) was crushed and subjected to three sequential extractions, each utilizing 1000 mL of an aqueous solution adjusted to pH 7.0 ± 0.1 with NaOH. The supernatants obtained through centrifugation were acidified to a pH of 3.5 using hydrochloric acid (HCl) and then chilled at 4°C for 12 hours. The precipitate obtained from subsequent centrifugation (12,000 rpm, 30 min) was redissolved after adjusting the pH back to 7.0, followed by ultrafiltration through a membrane with a 3000 Da molecular weight cut-off (MWCO) at 4500 rpm for 60 minutes. The retained fraction (>3 kDa) was collected and lyophilized to obtain purified AGP powder, which was stored at −20 °C until further use. The protein content was determined by the Bradford assay, and the purity of the preparation was calculated as the percentage of protein content relative to the total dry weight of the lyophilized powder..The polysaccharide content was determined by the phenol–sulfuric acid method with glucose as the standard, and the polysaccharide purity was 18.40% ± 0.91%.. The physicochemical properties of AGP were assessed across three independently prepared batches to evaluate batch-to-batch reproducibility

**Structural characterization**

*Amino acid analysis*

AGP (30 mg) was weighed into a hydrolysis tube, followed by the addition of 10 mL of 6 mol/L HCl solution. The mixture was thoroughly mixed and vortexed. Hydrolysis was conducted at 110 °C for 24 hours. Once hydrolysis was complete, the hydrolyzate was concentrated to dryness under vacuum. Sodium citrate buffer was subsequently added, and the mixture was subjected to ultrasonic cleaning for thorough mixing. Afterward, the solution was filtered through a 0.22 μm microporous filter membrane. Finally, the sample solutions were analyzed using an automatic amino acid analyzer.

*UV-Vis spectroscopy detection*

AGP lyophilized powder (1 mg) was dissolved in distilled water (pH 7.0) to yield a final concentration of 2 mg/mL. UV-Vis spectra were recorded using a Shimadzu UV-1900 UV spectrophotometer, with the scanning range set from 250 to 500 nm and a scan speed of 60 nm/min.

*Fourier transform infrared (FT-IR) detection*

AGP and KBr were mixed thoroughly and ground to a completely homogeneous powder. The resulting mixture was then pressed into a transparent pellet. Fourier-transform infrared (FTIR) measurements were performed on the transparent pellet using a Bruker Vertex 70 V spectrometer (Bruker Optics, Germany), with a scanning range of 500 to 4000 $cm^{-1}$, 64 scans, and a resolution of 4 $cm^{-1}$.

*Intrinsic fluorescence spectroscopy detection*

First, AGP freeze-dried powder was dissolved in the designated solution to achieve a final concentration of 0.5 mg/mL. Subsequently, the protein fluorescence spectrum of the resulting solution was determined using a fluorescence spectrophotometer, with the instrument parameters

set as follows: excitation wavelength fixed at 280 nm, emission wavelength scanned from 300 to 550 nm, and slit width adjusted to 5 nm.

*Circular dichroism (CD) spectroscopy detection*

CD spectroscopy was utilized to investigate the secondary structure of the protein in the far-ultraviolet region. CD spectra of AGP solutions at pH 5.0, 7.0, and 9.0 were recorded on a Jasco J-1500 spectropolarimeter, with the following instrumental parameters: scanning range of 190–260 nm, scan speed of 100 nm/min, bandwidth of 1.0 nm, response time of 0.5 s, and data interval of 1.0 nm.

*Two-dimensional electrophoresis (2-DE) of AGP*

Purified AGP sample was separated via two-dimensional electrophoresis. The six most abundant protein bands, spanning a molecular weight range of 10–75 kDa, were excised for subsequent identification. The gel pieces were subjected to in-gel enzymatic digestion, and the resulting peptides were analyzed using high-resolution mass spectrometry. Protein identification was based on multiple parameters, including the number of matched peptides, protein score, and molecular weight. The final assignment for each band was determined by combining the highest peptide matching scores with their practical relevance.

## Results

### Extraction, physicochemical and structural characterization of AGP

AGP was isolated from American ginseng roots, achieving an extraction yield of 2.59% and a protein purity of 81.42% ± 1.08%. The non-protein fraction mainly consisted of residual polysaccharides (18.40%±0.91 of the lyophilized powder), as determined by the phenol‑sulfuric acid method （Table S1）.The protein component was further characterized using various analytical techniques, including amino acid composition analysis, Fourier-transform infrared (FT-IR) spectroscopy, ultraviolet-visible (UV-Vis) spectroscopy, intrinsic fluorescence spectroscopy, circular dichroism (CD), and two-dimensional electrophoresis (2-DE).

Amino acid composition analysis (Fig. S2A) revealed AGP contains 17 distinct amino acids, with a total content of 83.57 g/100 g. Aspartic acid (Asp) and leucine (Leu) were the most abundant residues, accounting for 12.41% and 11.78% of the total amino acid content, respectively, followed by glutamic acid (Glu, 9.85%) and phenylalanine (Phe, 8.22%). Notably, essential amino acids (including histidine) constituted 41.07 g/100 g. Hydrophobic amino acids (39.39 g/100 g) dominated the composition, while acidic (16.69 g/100 g) and basic (14.93 g/100 g) amino acids were present in AGP. AGP also contained significant levels of aromatic (11.53 g/100 g), branched-chain (18.42 g/100 g), and sulfur-containing (2.17 g/100 g) amino acids, which are critical for its potential bioactivity and structural stability.

UV-Vis spectroscopy (Fig. S2B) showed a characteristic absorption maximum at 280 nm, consistent with the presence of aromatic amino acid residues (tryptophan, tyrosine, and phenylalanine). This finding correlated directly with the amino acid composition data, where Phe and Tyr collectively accounted for 11.53 g/100 g of AGP. FT-IR analysis (Fig. S2C) confirmed the typical protein secondary structure of AGP: a broad band at 3274 $cm^{-1}$ indicated O–H stretching vibrations from bound water and hydroxyl groups; a peak at 2921 $cm^{-1}$ corresponded to asymmetric stretching of aliphatic $–CH_2–$ groups; and amide I (1634 $cm^{-1}$), amide II (1527 $cm^{-1}$), and amide III (1239 $cm^{-1}$) bands validated the presence of peptide bonds. A distinct peak at 1392 $cm^{-1}$, attributed to symmetric stretching of free carboxylate ($COO^-$) groups, aligned with the high

content of acidic amino acids (Asp and Glu) identified in the composition analysis.

Intrinsic fluorescence spectroscopy was utilized to assess AGP's tertiary structure stability under varying pH conditions (Fig. S2D). Upon excitation at 280 nm, the maximum emission wavelength ($\lambda_{max}$) remained consistent at 338–339 nm across pH 5.0, 7.0, and 9.0, with no significant red shift observed. This indicates that the microenvironment surrounding the aromatic amino acid residues in AGP is highly stable, suggesting robust tertiary structure integrity under both acidic and alkaline conditions. CD spectroscopy (Fig. S2E) further characterized the secondary structure of AGP, revealing a strong positive band at 195 nm and a distinct negative band near 205 nm, which are signature features of β-sheet-rich proteins. Deconvolution of the CD spectra estimated that β-sheets constituted 56.6% of the secondary structure, with α-helices (14.7%), random coils (9.3%), and turns (19.4%) accounting for the remainder. This structural profile aligns with the amino acid composition, as AGP is enriched in residues (threonine, tyrosine, isoleucine, and phenylalanine) that favor β-sheet formation.

The purified AGP was analyzed using 2-DE, resulting in a complex protein profile (Fig. S2F). Most of the highly abundant protein species were found between 10-75 kDa in molecular weight. To identify the key components, the six most intense protein spots (indicated in Fig. S2F) were excised and identified by MALDI-TOF/TOF MS. The identification results, including protein names, accession numbers, peptide count, molecular weight, and score values, are summarized in Table S3. The six major proteins were identified as RNase-like major storage protein, major latex-like protein, phosphoglycerate kinase, glyceraldehyde-3-phosphate dehydrogenase, pathogenesis-related protein 10, and ribonuclease.

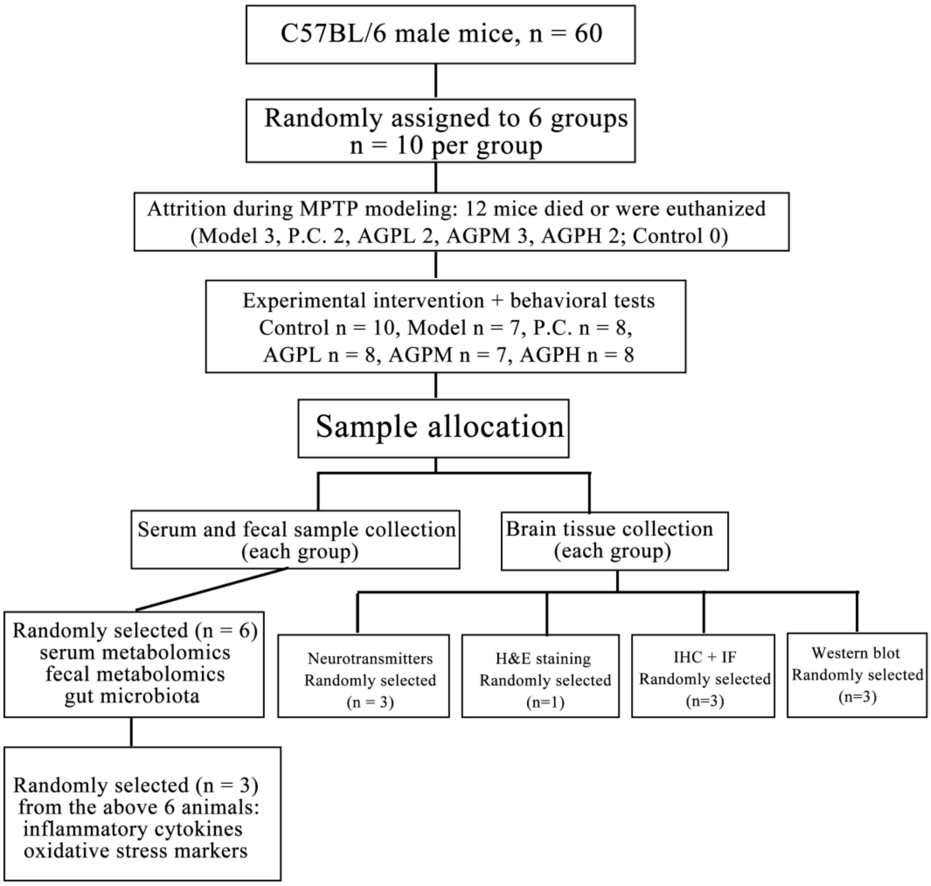


**Fig. S1 Flowchart of the experimental design and sample allocation.**

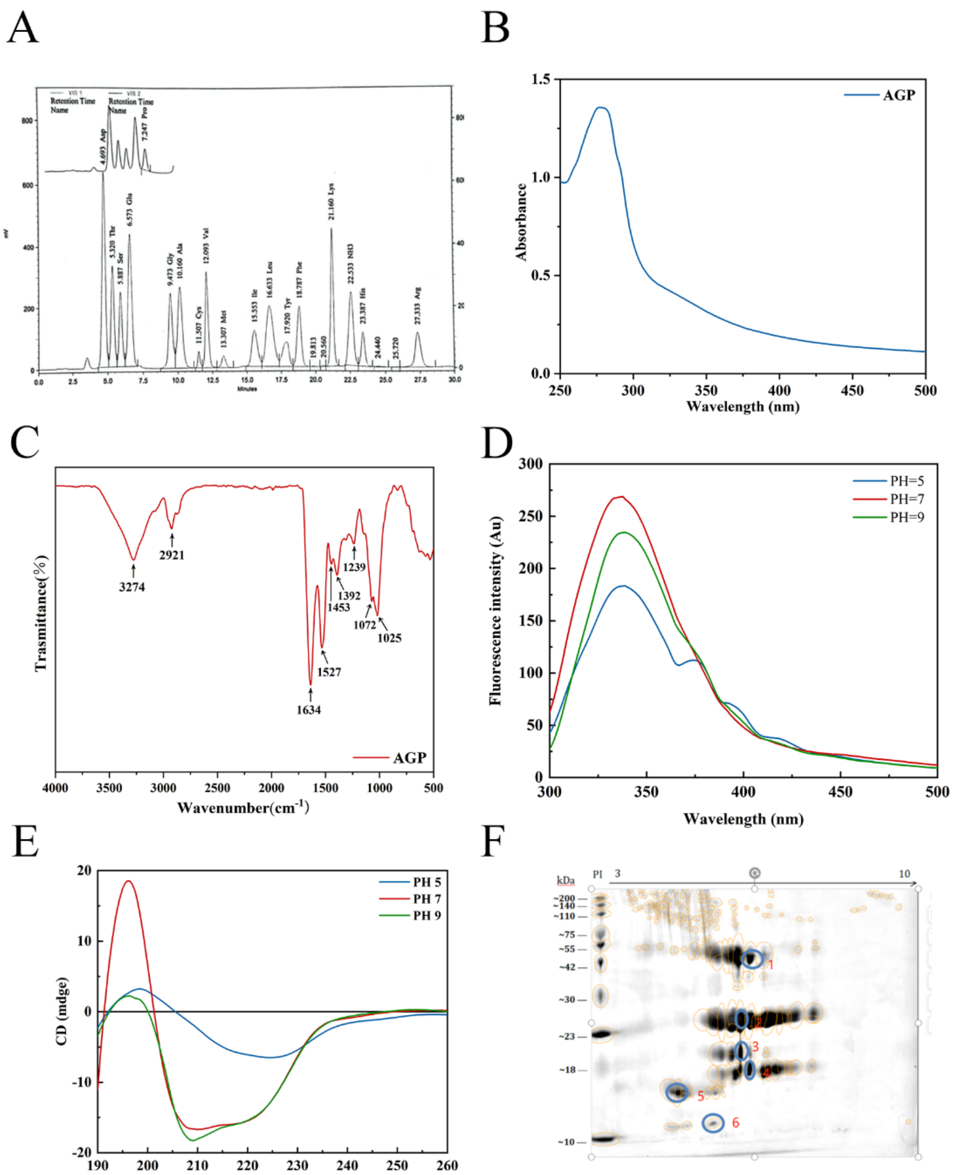


**Fig. S2** Characterization of AGP structure. (A) Amino Acid Composition. (B) UV absorption spectrum. (C) FTIR spectrum. (D) Intrinsic fluorescence spectrum. (E) CD spectrum (190-260 nm). (F) 2-DE electrophoresis map.

**Table S1.** Preparation yield of AGP

| Sample No. | Raw material (g) | Lyophilized powder (g) | Lyophilized powder yield (%) | Lyophilized concentration (mg/mL) | Protein content (mg/mL) | Protein purity (%) |
|---|---|---|---|---|---|---|
| 1 | 30.13 | 0.94 | 3.11 | 0.59 | 0.47 | 80.01 |
| 2 | 30.05 | 0.89 | 2.97 | 0.52 | 0.43 | 82.62 |
| 3 | 30.08 | 0.92 | 3.07 | 0.58 | 0.47 | 81.64 |
| Mean | 30.08 | 0.92 | 3.05 | 0.56 | 0.46 | 81.42 |
| SD | 0.03 | 0.02 | 0.06 | 0.03 | 0.02 | 1.08 |

**Table S2.** Mean body weights and working solution protein concentrations for AGP-treated groups during the treatment period.

| Week | AGPL mean body weight (g) | AGPL working concentration (mg/mL) | AGPM mean body weight (g) | AGPM working concentration (mg/mL) | AGPH mean body weight (g) | AGPH working concentration (mg/mL) |
|---|---|---|---|---|---|---|
| 1 | 21.94 | 4.39 | 21.93 | 8.77 | 22.19 | 13.32 |
| 2 | 22.66 | 4.53 | 22.77 | 9.11 | 23.17 | 13.9 |
| 3 | 23.34 | 4.67 | 23.36 | 9.34 | 23.21 | 13.92 |
| 4 | 24.25 | 4.85 | 24.71 | 9.89 | 24.31 | 14.58 |

**Table S3.** Identification of six protein bands from AGP

| Sample No. | Protein Name | Molecular Weight (kDa) | Peptide Count | Amino Acid Count |
|---|---|---|---|---|
| 2 | Ribonuclease-like major storage protein | 27.468 | 23 | 238 |
| 5 | Major latex-like protein | 16.871 | 6 | 151 |
| 1 | Phosphoglycerate kinase (PGK1) | 42.322 | 16 | 337 |
| 3 | Glyceraldehyde-3-phosphate dehydrogenase (GAPDH) | 22.303 | 11 | 337 |
| 4 | Pathogenesis-related protein 10 (PR-10) | 17.773 | 6 | 158 |
| 6 | Ribonuclease 2 (RNase 2) | 16.492 | 3 | 154 |

**Table S4.** Secondary structure content of AGP at different pH values

| Secondary structure | pH 5 | pH 7 | pH 9 |
|---|---|---|---|
| α-helix | 32.7% | 14.7% | 16.3% |
| β-sheet | 0 | 56.6% | 36.4% |
| β-turn | 36.8% | 9.3% | 17.4% |
| Random coil | 30.5% | 19.4% | 29.9% |

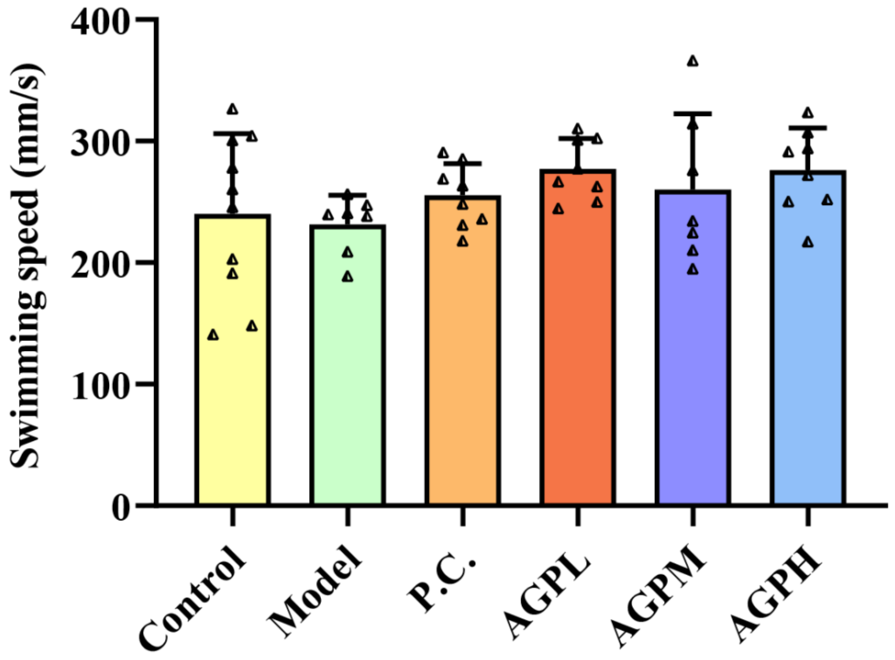


**Fig. S3.** Swimming speed in the Morris water maze test.

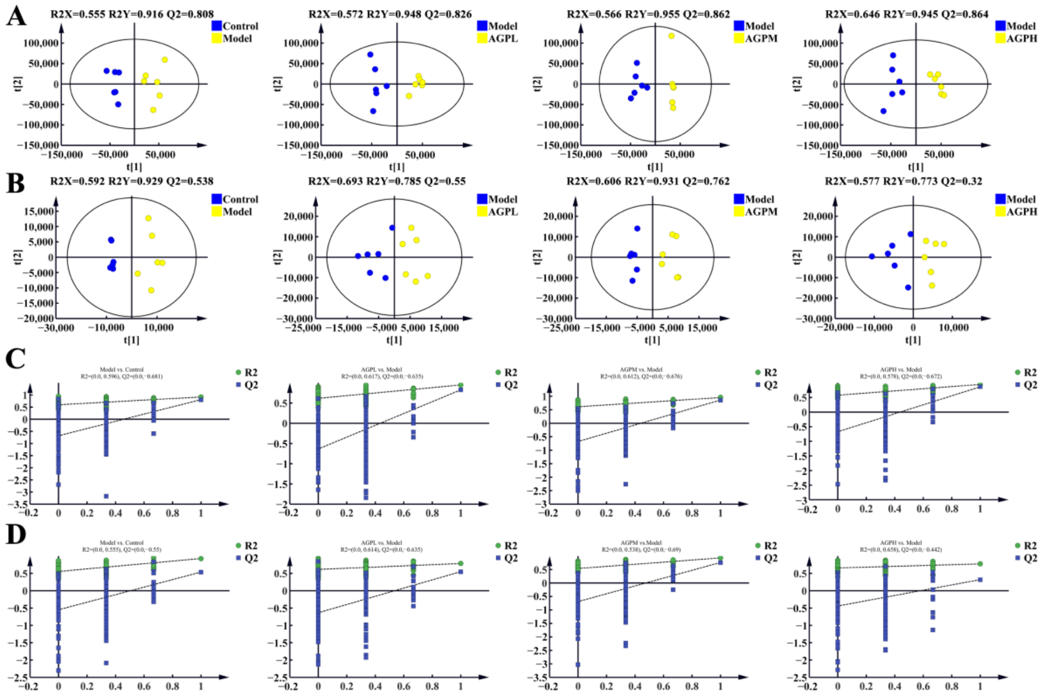


**Fig. S4.** Serum metabolite alterations in PD mice. (A) OPLS-DA score plots (Positive ion mode). (B) OPLS-DA score plots (Negative ion mode). (C) Permutation test plot (Positive ion mode, 200 Permutations). (D) Permutation test plot (Negative ion mode).

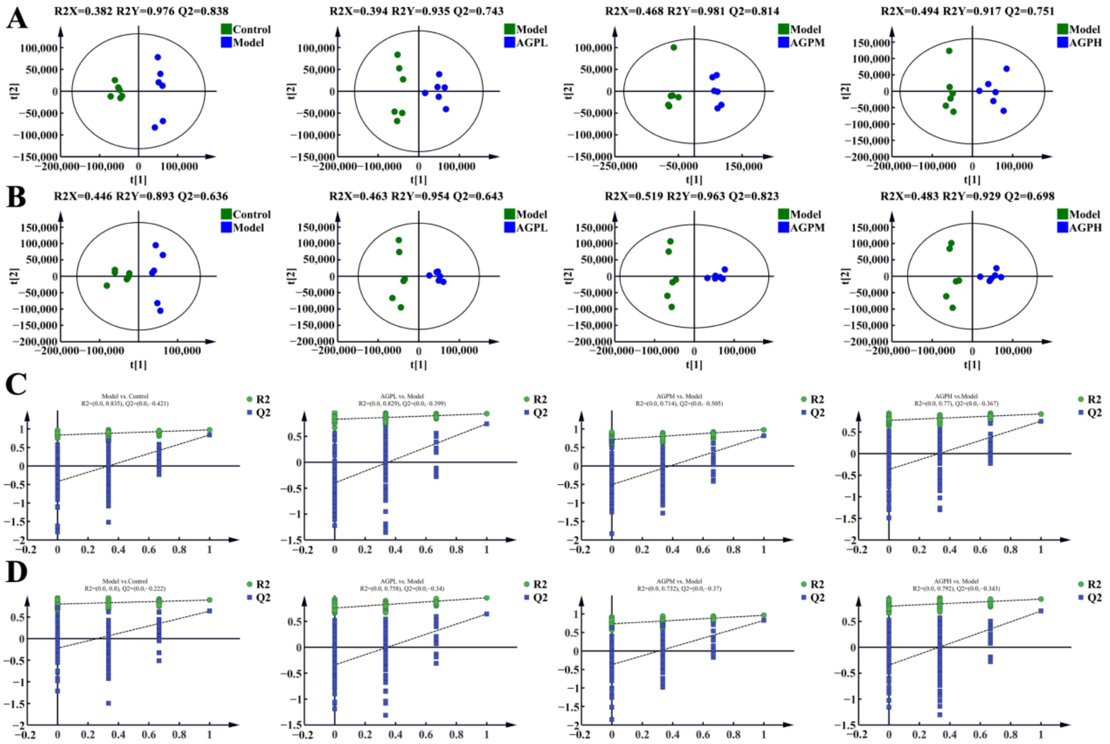


**Fig. S5**. Fecal metabolite alterations in PD mice. (A) OPLS-DA score plots (Positive ion mode). (B) OPLS-DA score plots (Negative ion mode). (C) Permutation test plot (Positive ion mode, 200 Permutations). (D) Permutation test plot (Negative ion mode).

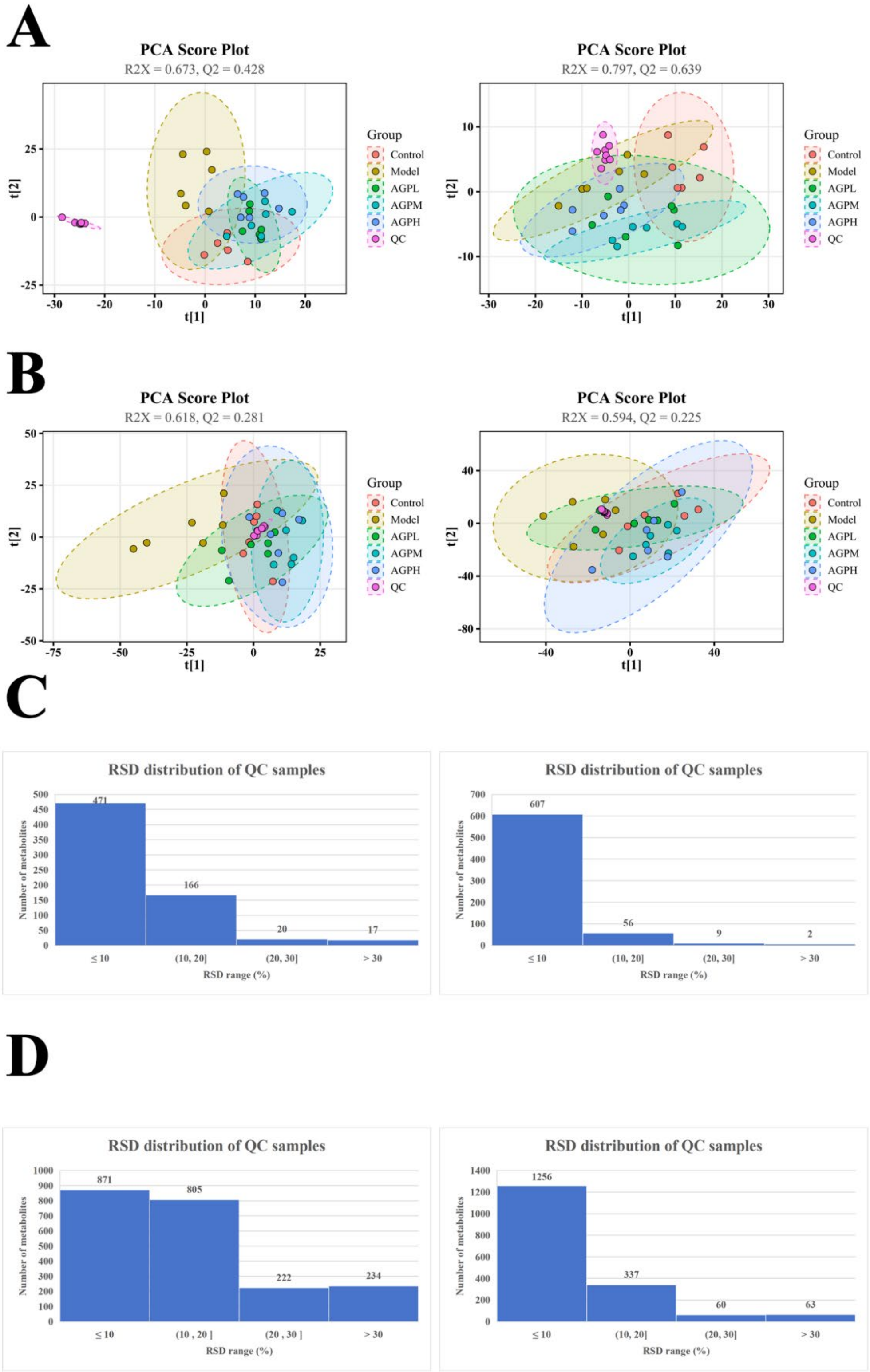


**Fig. S6.** PCA clustering and QC assessment of untargeted metabolomics data. (A,B) PCA plots of serum (A) and fecal (B) metabolomes derived from positive and negative ion modes. Separate 95% confidence ellipses were generated for each group (different from the global ellipse in the main text) to illustrate group-based sample clustering. (C,D) RSD distribution of metabolic features in

QC samples. The x-axis presents RSD intervals (<10%, 10% – 20%, 20% – 30%, >30%), and the y-axis shows the number of corresponding features for serum (C) and feces (D).

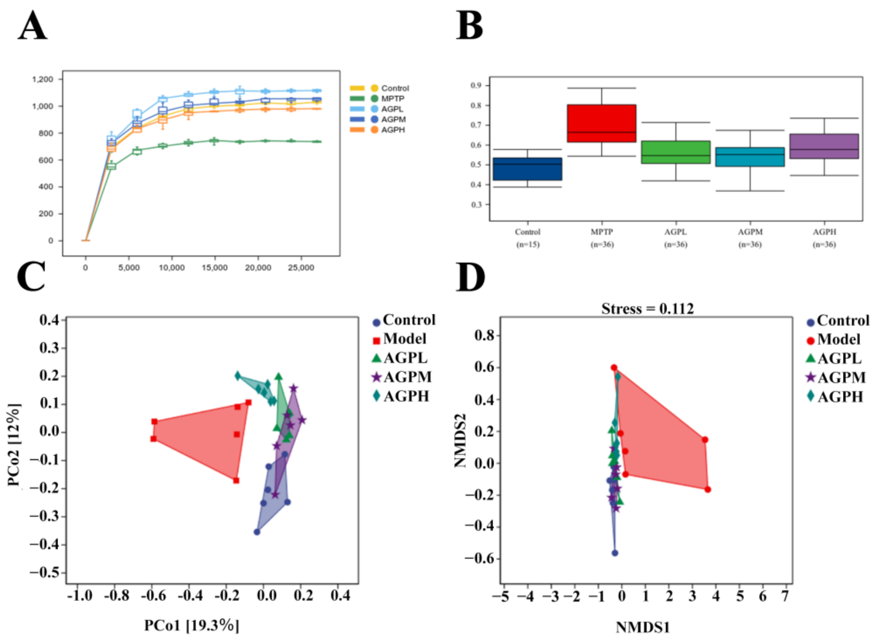


**Fig. S7**. Sequencing saturation and bacterial community structural analysis. (A) Rarefaction curves reflecting sequencing depth and species saturation across samples. (B) PERMANOVA pairwise comparison results of microbial community dissimilarity. (C) PCoA plot based on Bray–Curtis distance matrices. (D) NMDS analysis.

**Table S5.** Statistics of sequence quality control and filtering procedures for 16S rRNA gene sequencing

| SampleID | Input | Filtered | Denoised | Merged | Non-chimeric | Non-singleton |
|---|---|---|---|---|---|---|
| Control1 | 98281 | 93422 | 91202 | 78668 | 45082 | 44963 |
| Control2 | 94459 | 89810 | 87701 | 76376 | 50399 | 50289 |
| Control3 | 97494 | 92591 | 89754 | 75673 | 58131 | 58084 |
| Control4 | 99881 | 94835 | 92329 | 79523 | 50232 | 50122 |
| Control5 | 96605 | 91730 | 89377 | 76912 | 49526 | 49425 |
| Control6 | 97083 | 92107 | 90483 | 80606 | 47459 | 47358 |
| Model1 | 86682 | 82958 | 81250 | 72739 | 49990 | 49947 |
| Model2 | 80616 | 76680 | 75079 | 65253 | 40068 | 39991 |
| Model3 | 61731 | 58808 | 57282 | 48951 | 36514 | 36460 |
| Model4 | 69863 | 66039 | 64257 | 54317 | 31704 | 31583 |
| Model5 | 92952 | 87888 | 85642 | 73667 | 43207 | 43105 |
| Model6 | 87577 | 83364 | 81678 | 72179 | 56619 | 56593 |
| AGPL1 | 101137 | 97239 | 95656 | 86546 | 51592 | 51483 |
| AGPL2 | 98219 | 94184 | 91724 | 77892 | 43779 | 43644 |
| AGPL3 | 122079 | 115779 | 113509 | 100410 | 60629 | 60510 |

| | | | | | | |
|---|---|---|---|---|---|---|
| AGPL4 | 125849 | 119679 | 116702 | 99504 | 54006 | 53762 |
| AGPL5 | 114460 | 108670 | 105714 | 89753 | 41883 | 41568 |
| AGPL6 | 132552 | 125989 | 124816 | 116859 | 75128 | 75091 |
| AGPM1 | 91867 | 87973 | 85117 | 71579 | 53701 | 53651 |
| AGPM2 | 87454 | 83975 | 82241 | 74007 | 47140 | 47103 |
| AGPM3 | 84606 | 81075 | 78013 | 63786 | 37396 | 37240 |
| AGPM4 | 126413 | 120117 | 117802 | 104404 | 74011 | 73934 |
| AGPM5 | 121533 | 114984 | 113058 | 100769 | 69953 | 69879 |
| AGPM6 | 105488 | 100029 | 97639 | 84857 | 51577 | 51394 |
| AGPH1 | 80872 | 77412 | 75246 | 63452 | 44390 | 44285 |
| AGPH2 | 57580 | 55242 | 53692 | 45581 | 31758 | 31701 |
| AGPH3 | 73500 | 70592 | 68704 | 58461 | 36199 | 36111 |
| AGPH4 | 129675 | 122988 | 121046 | 108499 | 83394 | 83350 |
| AGPH5 | 121206 | 114952 | 111175 | 90628 | 49047 | 48813 |
| AGPH6 | 118645 | 112781 | 111277 | 101557 | 77467 | 77428 |

**Table S6.** Pairwise PERMANOVA analysis of bacterial community structure based on Bray–Curtis distance

| Group1 | Group2 | Sample size | Permutations | pseudo-F | p-value | q-value |
|---|---|---|---|---|---|---|
| all | - | 30 | 999 | 2.9003 | 0.001 | - |
| Control | MPTP | 12 | 999 | 3.0090 | 0.002 | 0.0033 |
| Control | AGPL | 12 | 999 | 3.0592 | 0.001 | 0.0033 |
| Control | AGPM | 12 | 999 | 2.6875 | 0.006 | 0.0067 |
| Control | AGPH | 12 | 999 | 3.8538 | 0.002 | 0.0033 |
| MPTP | AGPL | 12 | 999 | 3.2450 | 0.002 | 0.0033 |
| MPTP | AGPM | 12 | 999 | 3.3918 | 0.002 | 0.0033 |
| MPTP | AGPH | 12 | 999 | 2.3400 | 0.005 | 0.0062 |
| AGPL | AGPM | 12 | 999 | 2.0068 | 0.011 | 0.0110 |
| AGPL | AGPH | 12 | 999 | 2.6952 | 0.001 | 0.0033 |
| AGPM | AGPH | 12 | 999 | 2.4708 | 0.005 | 0.0062 |

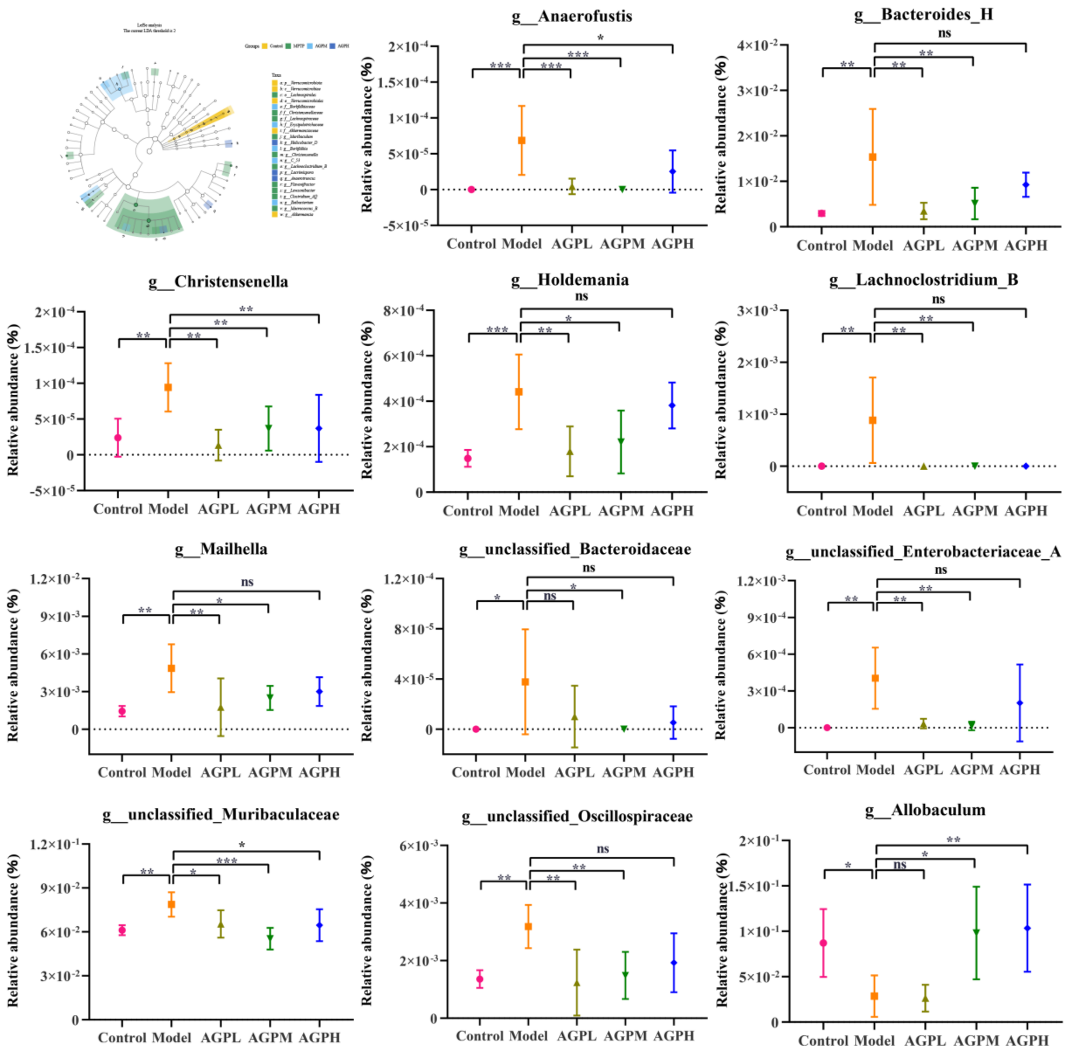


**Fig. S8.** Linear discriminant analysis effect size (LEfSe) analysis and relative abundance of significantly differential bacterial genera among MPTP-induced PD mice, control group and AGP treatment groups.

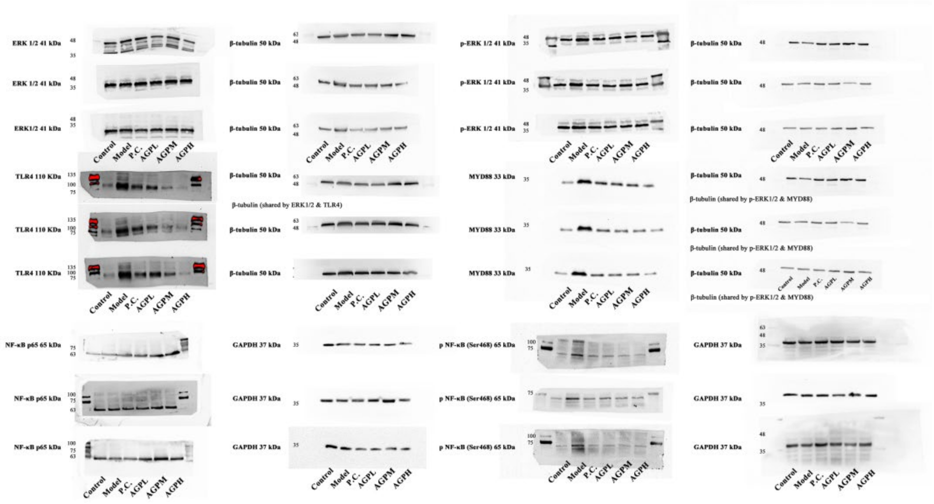


**Fig. S9.** Original, uncropped western blot membranes.

**Table S7.** Trends of differential metabolites in serum experimental groups

| Metabolites | Formula | Classification | MPTP vs.Control | AGP vs.MPTP | Fragments(m/z) |
|---|---|---|---|---|---|
| Tridecanol | C13H28O | Long-chain fatty alcohol | Down | UP | 57.0699，85.1017，111.1174，69.0704 |
| N-Hexadecanoyl-serine | C19H37NO4 | Fatty amide | UP | Down | 57.0699，71.0855，85.1012，95.0855，109.1012 |
| 3-Hydroxyicosanoic Acid | C20H40O3 | Fatty acid | UP | Down | 71.0855 |
| Sphinganine | C18H39NO2 | Organic compounds | UP | Down | 57.0709，83.0889，95.0850，109.1024，123.1158，69.0703 |
| 12-HETE | C20H32O3 | Fatty acid | UP | Down | 257.2275，283.2067，301.2173，319.2279，59.0139，135.1179，139.1128，153.1285，163.1128179.1078 |
| LysoPC | C24H50NO7P | Organic compounds | UP | Down | 255.2329,78.9591,152.9958,257.2486,391.2255 |
| 3-Phenylpropionylglycine | C11H13NO3 | Amino acids | Down | UP | 59.0139 |

**Table S8.** Statistical parameters of differential metabolites in serum (Model vs. Control).

| M/Z-RT | VIP | FC | LOG2 | P | FDR | Metabolites |
|---|---|---|---|---|---|---|
| 200.2374-5.88 | 10.95 | 0.6507 | -0.6200 | 0.0398 | 0.1387 | Tridecanol |
| 371.3156-15.29 | 4.43 | 1.6241 | 0.6997 | 0.0003 | 0.0357 | / |
| 344.2798-10.28 | 3.63 | 2.9408 | 1.5562 | 0.0009 | 0.0357 | N-Hexadecanoyl-serine |
| 274.2741-7.03 | 3.41 | 1.2020 | 0.2655 | 0.0329 | 0.1233 | 3-Hydroxyicosanoic Acid |
| 256.2635-11.99 | 2.37 | 1.4438 | 0.5299 | 0.0088 | 0.0683 | / |
| 362.3265-7.15 | 2.26 | 1.4102 | 0.4959 | 0.0027 | 0.0508 | / |
| 346.3316-8.13 | 1.61 | 1.3842 | 0.4691 | 0.0038 | 0.0556 | / |
| 284.2948-13.62 | 1.08 | 1.3113 | 0.3910 | 0.0372 | 0.1339 | Sphinganine |
| 319.2845-10.98 | 1.20 | 1.9003 | 0.9262 | 0.0011 | 0.0366 | / |
| 270.3154-8.07 | 9.89 | 0.8136 | -0.2976 | 0.0051 | 0.0668 | / |
| 354.3003-13 | 9.76 | 1.8341 | 0.8751 | 0.0084 | 0.0683 | / |
| 227.0638-7.51 | 1.54 | 2.0656 | 1.0466 | 0.0091 | 0.0683 | / |
| 295.2269-10.28 | 1.40 | 2.4363 | 1.2847 | 0.0014 | 0.0459 | / |
| 316.9477-0.33 | 2.2228 | 0.6608 | -0.5977 | 0.0072 | 0.0257 | / |
| 319.2283-9.77 | 1.1768 | 1.5317 | 0.6151 | 0.0201 | 0.0556 | 12-HETE |
| 1035.6647-9.96 | 3.2932 | 2.1106 | 1.0777 | 0.0038 | 0.0145 | LysoPC |
| 266.1038-7.92 | 1.7820 | 0.7458 | -0.4232 | 0.0038 | 0.0145 | 3-Phenylpropionylglycine |
| 612.3318-9.41 | 1.4538 | 1.4482 | 0.5343 | 0.0161 | 0.0468 | LysoPE |

**Table S9.** Statistical parameters of differential metabolites in serum (AGPL vs. Model).

| M/Z-RT | VIP | FC | LOG2 | P | FDR | Metabolites |
|---|---|---|---|---|---|---|
| 200.2374-5.88 | 12.1004 | 1.6051 | 0.6826 | 0.0080 | 0.0369 | Tridecanol |
| 371.3156-15.29 | 3.4100 | 0.7326 | -0.4490 | 0.0185 | 0.0659 | / |
| 344.2798-10.28 | 2.4294 | 0.6071 | -0.7201 | 0.0140 | 0.0546 | N-Hexadecanoyl-serine |
| 274.2741-7.03 | 3.4756 | 0.8078 | -0.3080 | 0.0153 | 0.0572 | 3-Hydroxyicosanoic Acid |
| 256.2635-11.99 | 3.2127 | 0.5815 | -0.7822 | 0.0011 | 0.0079 | / |
| 362.3265-7.15 | 2.1465 | 0.7088 | -0.4966 | 0.0028 | 0.0174 | / |
| 346.3316-8.13 | 1.9076 | 0.6736 | -0.5700 | 0.0072 | 0.0352 | / |
| 284.2948-13.62 | 1.6213 | 0.6276 | -0.6720 | 0.0005 | 0.0052 | Sphinganine |
| 319.2845-10.98 | 1.2356 | 0.5341 | -0.9047 | 0.0018 | 0.0122 | / |
| 319.2283-9.77 | 1.2088 | 0.6017 | -0.7328 | 0.0124 | 0.1200 | 12-HETE |

**Table S10.** Statistical parameters of differential metabolites in serum (AGPM vs. Model)

| M/Z-RT | VIP | FC | LOG2 | P | FDR | Metabolites |
|---|---|---|---|---|---|---|
| 371.3156-15.29 | 5.1853 | 0.6342 | -0.6570 | 0.0006 | 0.0058 | / |
| 344.2798-10.28 | 4.1117 | 0.3486 | -1.5205 | 0.0011 | 0.0084 | N-Hexadecanoyl-serine |
| 346.3316-8.13 | 1.4274 | 0.7958 | -0.3296 | 0.0311 | 0.0926 | / |
| 319.2845-10.98 | 1.4488 | 0.5123 | -0.9650 | 0.0008 | 0.0069 | / |
| 270.3154-8.07 | 14.5281 | 1.2972 | 0.3754 | 0.0011 | 0.0081 | / |
| 354.3003-13 | 11.3278 | 0.5236 | -0.9334 | 0.0074 | 0.0300 | / |
| 227.0638-7.51 | 1.3411 | 0.6391 | -0.6458 | 0.0417 | 0.1178 | / |
| 295.2269-10.28 | 1.6272 | 0.3839 | -1.3813 | 0.0016 | 0.0103 | / |
| 1035.6647-9.96 | 3.2850 | 0.6425 | -0.6382 | 0.0221 | 0.0713 | LysoPC |
| 266.1038-7.92 | 2.1797 | 1.3372 | 0.4192 | 0.0042 | 0.0261 | 3-Phenylpropionylglycine |

**Table S11.** Statistical parameters of differential metabolites in serum (AGPH vs. Model).

| M/Z-RT | VIP | FC | LOG2 | P | FDR | Metabolites |
|---|---|---|---|---|---|---|
| 200.2374-5.88 | 8.7691 | 1.4243 | 0.5102 | 0.0228 | 0.0834 | Tridecanol |
| 344.2798-10.28 | 3.0848 | 0.4077 | -1.2945 | 0.0027 | 0.0173 | N-Hexadecanoyl-serine |
| 256.2635-11.99 | 2.9478 | 0.5898 | -0.7617 | 0.0001 | 0.0011 | / |
| 284.2948-13.62 | 1.5338 | 0.6396 | -0.6448 | 0.0004 | 0.0032 | Sphinganine |
| 270.3154-8.07 | 9.3819 | 1.2237 | 0.2913 | 0.0022 | 0.0155 | / |
| 354.3003-13 | 9.1871 | 0.4997 | -1.0008 | 0.0067 | 0.0307 | / |
| 295.2269-10.28 | 1.2487 | 0.4141 | -1.2721 | 0.0025 | 0.0159 | / |

**Table S12.** Trends of differential metabolites in feces experimental groups

| NO. | M/Z | RT | Metabolites | Formula | Classification | MPTP vs. Control | AGP vs. MPTP | Frajment |
|---|---|---|---|---|---|---|---|---|
| 1 | 228.0505 | 2.45 | pipecolic acid betaine | C21H20O10 | Organic compou | UP | Down | 139.0390 |

| | | | | | | | | |
|---|---|---|---|---|---|---|---|---|
| | | | | | nds | | | |
| 2 | 389.2690 | 4.57 | 14-HDoHE | C23H32O3 | Fatty acid | Down | UP | 145.1012，97.0648，259.1693 |
| 3 | 121.0294 | 3.54 | Benzoic acid | C7H6O2 | Background contaminant | Down | UP | 121.0295，95.0133 |
| 4 | 190.0509 | 3.55 | Indole-3-carboxaldehyde | C9H7NO | Organic compounds | Down | UP | 144.0449，116.0518 |
| 5 | 226.0358 | 2.46 | Quinolinic acid | C7H5NO4 | Organic compounds | UP | Down | 71.0133 |
| 6 | 243.1237 | 3.55 | 10-hydroxydodec-5-enedioic acid | C12H20O5 | Fatty acid | Down | UP | 183.1027，243.1238，59.0138，225.1132 |
| 7 | 407.2801 | 5.15 | Ursocholic acid | C24H40O5 | Bile acid | Down | UP | 407.2803，105.1172，291.1966 |
| 8 | 453.2863 | 4.78 | 11-Hydroxyeicosatetraenoate glyceryl ester | C23H38O5 | Fatty acid | Down | UP | 59.0138 |

**Table S13.** Statistical parameters of differential metabolites in feces (Model vs. Control)

| M/Z-RT (min) | VIP | FC(M） | log2(M) | P-VALUE | FDR | Metabolites |
|---|---|---|---|---|---|---|
| 270.32-6.93 | 9.6727 | 3.2638 | 1.7065 | 0.0002 | 0.004747462 | / |
| 146.12-1.27 | 1.8301 | 0.4548 | -1.1367 | 0.0039 | 0.02975452 | / |
| 316.28-6.11 | 2.7810 | 14.6960 | 3.8773 | 0.0015 | 0.03015057 | / |
| 160.13-1.9 | 1.0112 | 0.4948 | -1.0150 | 0.0076 | 0.048181091 | / |
| 333.13-3.46 | 1.6427 | 0.5792 | -0.7880 | 0.0079 | 0.079762384 | / |
| 243.12-3.73 | 1.2825 | 0.4721 | -1.0827 | 0.0034 | 0.08177585 | / |
| 270.32-7.24 | 6.9457 | 6.4925 | 2.6988 | 0.0057 | 0.083147395 | / |
| 245.14-3.55 | 1.6732 | 0.5348 | -0.9029 | 0.0300 | 0.096417763 | / |

| | | | | | | |
|---|---|---|---|---|---|---|
| 243.09-4.19 | 1.6526 | 0.2567 | -1.9617 | 0.0052 | 0.096417763 | Diferuloylputrescine |
| 121.1-3.55 | 1.3133 | 0.5492 | -0.8646 | 0.0306 | 0.109816938 | / |
| 487.31-5.63 | 1.7667 | 0.3541 | -1.4978 | 0.0002 | 0.122689156 | 25-Hydroxyvitamin D3-26,23-lactone |
| 453.29-4.78 | 1.5191 | 0.4442 | -1.1709 | 0.0006 | 0.125661591 | 11-Hydroxyeicosatetraenoate glyceryl ester |
| 471.31-5.58 | 1.3739 | 0.3369 | -1.5698 | 0.0013 | 0.125661591 | / |
| 163.11-3.55 | 1.1165 | 0.5668 | -0.8192 | 0.0348 | 0.128249928 | / |
| 243.12-3.55 | 5.2632 | 0.5494 | -0.8640 | 0.0165 | 0.13159306 | 10-hydroxydodec-5-enedioic acid |
| 315.12-4.08 | 2.2406 | 0.7386 | -0.4372 | 0.0132 | 0.13159306 | / |
| 160.04-3.16 | 1.3234 | 0.8055 | -0.3120 | 0.0076 | 0.13159306 | / |
| 243.12-3.65 | 2.2407 | 0.5707 | -0.8092 | 0.0110 | 0.136620849 | / |
| 389.27-4.57 | 1.1426 | 0.6730 | -0.5713 | 0.0157 | 0.136862725 | 14-HDoHE |
| 457.37-6.7 | 2.3044 | 3.0583 | 1.6127 | 0.0012 | 0.13860977 | / |
| 393.2-4.78 | 1.8638 | 0.6440 | -0.6349 | 0.0308 | 0.148099278 | / |
| 361.22-3.75 | 1.5842 | 0.7594 | -0.3971 | 0.0089 | 0.148099278 | / |
| 121.03-3.54 | 1.1603 | 0.6082 | -0.7175 | 0.0008 | 0.148099278 | Benzoic acid |
| 571.29-7.74 | 2.3110 | 29.7830 | 4.8964 | 0.0027 | 0.153116001 | / |
| 191.11-3.55 | 1.3555 | 0.5893 | -0.7629 | 0.0450 | 0.15483922 | / |
| 498.29-5. | 1.5353 | 3.9099 | 1.9671 | 0.0109 | 0.155215117 | Tauroche |

| | | | | | | |
|---|---|---|---|---|---|---|
| 1 | | | | | | nodesoxy cholic acid |
| 454.35-5.78 | 1.3091 | 3.0318 | 1.6002 | 0.0206 | 0.160040281 | / |
| 475.34-5.61 | 1.4349 | 0.4111 | -1.2825 | 0.0090 | 0.171997259 | / |
| 257.08-4.65 | 1.1856 | 2.3360 | 1.2240 | 0.0463 | 0.181849737 | / |
| 344.32-9.75 | 1.2583 | 0.2364 | -2.0806 | 0.0036 | 0.184766084 | / |
| 439.36-6.7 | 1.4006 | 1.9509 | 0.9642 | 0.0046 | 0.188858825 | / |
| 357.28-6.57 | 2.1242 | 0.4422 | -1.1773 | 0.0484 | 0.188858825 | / |
| 437.29-6.11 | 1.9777 | 0.4292 | -1.2204 | 0.0046 | 0.189452308 | / |
| 471.31-6.04 | 1.2061 | 0.4980 | -1.0057 | 0.0338 | 0.189452308 | / |
| 159.08-0.81 | 1.2916 | 0.5960 | -0.7465 | 0.0311 | 0.194484168 | / |
| 149.05-0.83 | 1.8584 | 2.0400 | 1.0286 | 0.0289 | 0.198502018 | / |
| 623.28-3.91 | 1.1287 | 4.4003 | 2.1376 | 0.0042 | 0.199088519 | / |
| 453.34-6.09 | 1.8756 | 4.6902 | 2.2296 | 0.0018 | 0.202774727 | / |
| 391.29-6.11 | 2.1840 | 0.4170 | -1.2619 | 0.0098 | 0.203404779 | / |
| 245.14-4.17 | 1.9419 | 1.8698 | 0.9029 | 0.0080 | 0.203404779 | / |
| 471.35-6.09 | 3.7539 | 4.7124 | 2.2365 | 0.0024 | 0.207593586 | / |
| 571.29-7.87 | 1.4618 | 6.8127 | 2.7682 | 0.0002 | 0.211368517 | / |
| 162.06-3.16 | 2.6014 | 0.8079 | -0.3077 | 0.0063 | 0.211976878 | / |
| 487.31-4.77 | 1.7736 | 0.4289 | -1.2213 | 0.0157 | 0.213537369 | / |
| 471.31-7.23 | 1.4749 | 0.4828 | -1.0505 | 0.0189 | 0.213537369 | / |
| 705.4-5.13 | 2.1652 | 0.3749 | -1.4153 | 0.0005 | 0.220727249 | / |
| 457.37-6. | 2.3138 | 2.5988 | 1.3778 | 0.0021 | 0.22092183 | / |

| | | | | | | |
|---|---|---|---|---|---|---|
| 1 | | | | | | |
| 375.29-6.57 | 1.6131 | 0.5339 | -0.9055 | 0.0248 | 0.22092183 | / |
| 228.05-2.45 | 2.4688 | 2.7771 | 1.4736 | 0.0045 | 0.222254194 | pipecolic acid betaine |
| 190.05-3.55 | 1.3621 | 0.2625 | -1.9295 | 0.0380 | 0.222955475 | Indole-3-carboxaldehyde |
| 285.11-4.2 | 1.6519 | 0.6037 | -0.7282 | 0.0284 | 0.223404943 | / |
| 144.05-3.55 | 2.8424 | 0.2586 | -1.9512 | 0.0414 | 0.223743437 | / |
| 371.26-4.81 | 1.5316 | 3.0385 | 1.6034 | 0.0161 | 0.224016241 | / |
| 285.17-3.61 | 1.1955 | 0.7532 | -0.4089 | 0.0161 | 0.228550042 | / |
| 329.1-4.21 | 4.4148 | 0.5742 | -0.8003 | 0.0138 | 0.230344338 | / |
| 329.1-4.14 | 2.3264 | 0.5745 | -0.7997 | 0.0373 | 0.230344338 | / |
| 440.26-3.43 | 1.4227 | 3.8078 | 1.9289 | 0.0366 | 0.230344338 | / |
| 134.06-3.9 | 2.6425 | 0.1975 | -2.3403 | 0.0004 | 0.232884618 | / |
| 226.04-2.46 | 2.3313 | 2.9314 | 1.5516 | 0.0025 | 0.24017293 | Quinolinic acid |
| 533.35-6.08 | 1.4752 | 3.9053 | 1.9655 | 0.0163 | 0.24017293 | / |
| 575.27-3.88 | 1.3959 | 2.2573 | 1.1746 | 0.0124 | 0.24017293 | / |
| 163.04-3.84 | 1.3038 | 2.7273 | 1.4475 | 0.0053 | 0.24017293 | Phenylpyruvic acid |
| 307.09-3.25 | 1.2251 | 2.3011 | 1.2023 | 0.0382 | 0.24017293 | / |
| 327.09-4.02 | 1.8042 | 0.3367 | -1.5704 | 0.0157 | 0.240964584 | / |
| 183.03-2.46 | 1.6835 | 2.9083 | 1.5402 | 0.0031 | 0.240964584 | / |
| 149.03-2.91 | 1.2691 | 3.2420 | 1.6969 | 0.0349 | 0.240964584 | / |
| 407.28-5.15 | 1.4945 | 0.7326 | -0.4489 | 0.0405 | 0.24381726 | Ursocholic acid |
| 329.23-5. | 1.4878 | 0.2979 | -1.7470 | 0.0106 | 0.24381726 | / |

| | | | | | | |
|---|---|---|---|---|---|---|
| 76 | | | | | | |
| 489.36-6.09 | 1.5695 | 4.4392 | 2.1503 | 0.0060 | 0.247285035 | / |

**Table S14.** Statistical parameters of differential metabolites in feces (AGPL vs. Model)

| M/Z-RT (min) | VIP | FC(M） | log2(M) | P-Value | FDR | Metabolites |
|---|---|---|---|---|---|---|
| 389.27-4.57 | 1.8400 | 1.9176 | 0.9393 | 0.0157 | 0.200794048 | 14-HDoHE |
| 471.31-5.58 | 1.24297 | 2.6368 | 1.3988 | 0.0013 | 0.236345744 | / |

**Table S15.** Statistical parameters of differential metabolites in feces (AGPM vs. Model)

| M/Z-RT (min) | VIP | FC (M） | log2 (M) | P-VALUE | FDR | Metabolites |
|---|---|---|---|---|---|---|
| 121.03-3.54 | 1.2705 | 1.9116 | 0.9348 | 0.0008 | 0.046362804 | Benzoic acid |
| 146.12-1.27 | 1.8290 | 2.8342 | 1.5030 | 0.0039 | 0.051780348 | / |
| 160.13-1.9 | 1.0718 | 2.6185 | 1.3887 | 0.0076 | 0.051780348 | / |
| 329.1-4.21 | 4.4111 | 1.9197 | 0.9409 | 0.0138 | 0.054484343 | / |
| 285.11-4.2 | 1.6270 | 1.8236 | 0.8668 | 0.0284 | 0.054484343 | / |
| 243.12-3.65 | 2.5086 | 2.0040 | 1.0029 | 0.0110 | 0.059350185 | / |
| 316.28-6.11 | 1.9190 | 0.2137 | -2.2261 | 0.0015 | 0.060812956 | / |
| 453.29-4.78 | 1.5682 | 3.0591 | 1.6131 | 0.0006 | 0.060842481 | 11-Hydroxyeicosatetraenoate glyceryl ester |
| 243.12-3.55 | 5.6177 | 2.0774 | 1.0548 | 0.0165 | 0.066672335 | 10-hydroxydodec-5-enedioic acid |
| 393.2-4.78 | 1.3454 | 1.4511 | 0.5372 | 0.0308 | 0.084297332 | / |
| 315.12-4.08 | 2.1540 | 1.4310 | 0.5171 | 0.0132 | 0.08508759 | / |
| 389.27-4.57 | 1.1080 | 1.7109 | 0.7748 | 0.0157 | 0.102860385 | 14-HDoHE |

| | | | | | | |
|---|---|---|---|---|---|---|
| 329.1-4.14 | 2.3262 | 1.8583 | 0.8940 | 0.0373 | 0.103172763 | / |
| 243.12-3.73 | 1.0214 | 2.1251 | 1.0875 | 0.0034 | 0.103675402 | / |
| 257.08-4.65 | 1.1618 | 0.3078 | -1.7000 | 0.0463 | 0.107865725 | / |
| 160.04-3.16 | 1.1819 | 1.2870 | 0.3640 | 0.0076 | 0.109780685 | / |
| 285.17-3.61 | 1.2759 | 1.4729 | 0.5586 | 0.0161 | 0.110831108 | / |
| 571.29-7.87 | 1.4135 | 0.0032 | -8.2681 | 0.0002 | 0.11595992 | / |
| 327.09-4.02 | 1.7527 | 3.0540 | 1.6107 | 0.0157 | 0.116830685 | / |
| 245.14-3.55 | 1.4923 | 2.0802 | 1.0567 | 0.0300 | 0.119451639 | / |
| 459.38-9.76 | 2.2391 | 0.4135 | -1.2740 | 0.0169 | 0.123503407 | / |
| 441.37-9.76 | 4.5356 | 0.4292 | -1.2203 | 0.0251 | 0.124558127 | / |
| 333.26-5.27 | 2.2629 | 4.8034 | 2.2641 | 0.0029 | 0.126236569 | / |
| 121.1-3.55 | 1.1302 | 1.9638 | 0.9736 | 0.0306 | 0.126236569 | / |
| 159.08-0.81 | 1.5118 | 2.1858 | 1.1282 | 0.0311 | 0.126236569 | / |
| 274.09-0.84 | 1.1804 | 0.2581 | -1.9538 | 0.0415 | 0.126236569 | / |
| 457.37-6.1 | 2.0952 | 0.1880 | -2.4115 | 0.0021 | 0.128535711 | / |
| 455.35-7.78 | 1.0605 | 0.3727 | -1.4238 | 0.0045 | 0.130718623 | / |
| 440.26-3.43 | 1.3565 | 0.1267 | -2.9802 | 0.0366 | 0.132548501 | / |
| 307.09-3.25 | 1.4126 | 0.1909 | -2.3894 | 0.0382 | 0.133817241 | / |
| 541.34-3.05 | 1.9654 | 0.0940 | -3.4108 | 0.0044 | 0.135061226 | / |
| 229.14-5.31 | 2.0826 | 4.4496 | 2.1537 | 0.0021 | 0.138198654 | / |
| 270.32-7.24 | 5.0393 | 1.8831 | 0.9131 | 0.0057 | 0.144065359 | / |
| 307.19-7.73 | 1.8245 | 6.2032 | 2.6330 | 0.0000 | 0.144147122 | / |
| 228.05-2.45 | 2.1349 | 0.2720 | -1.8785 | 0.0045 | 0.14568880 | pipecolic |

| | | | | | | |
|---|---|---|---|---|---|---|
| | | | | | 1 | acid betaine |
| 191.11-3.55 | 1.2024 | 1.8543 | 0.8909 | 0.0450 | 0.145688801 | / |
| 457.37-6.7 | 1.8251 | 0.3294 | -1.6023 | 0.0012 | 0.147033039 | / |
| 135.03-1.29 | 4.7115 | 5.2415 | 2.3900 | 0.0380 | 0.150427996 | Threonic acid |
| 329.23-5.76 | 1.8237 | 3.9920 | 1.9971 | 0.0106 | 0.153394263 | / |
| 575.27-3.88 | 1.4069 | 0.2176 | -2.2006 | 0.0124 | 0.161649019 | / |
| 290.09-1.29 | 1.4081 | 0.3144 | -1.6695 | 0.0406 | 0.161938475 | / |
| 146.06-3.85 | 1.8694 | 9.9477 | 3.3144 | 0.0107 | 0.163810517 | / |
| 226.04-2.46 | 1.9679 | 0.3056 | -1.7103 | 0.0025 | 0.16647264 | Quinolinic acid |
| 361.22-3.75 | 1.2834 | 1.3401 | 0.4224 | 0.0089 | 0.16647264 | / |
| 651.41-5.64 | 2.0613 | 0.1160 | -3.1077 | 0.0382 | 0.169208396 | / |
| 183.03-2.46 | 1.4333 | 0.2997 | -1.7383 | 0.0031 | 0.171785482 | / |
| 438.26-7.54 | 2.3460 | 0.6218 | -0.6856 | 0.0034 | 0.176805797 | / |
| 371.26-4.81 | 1.1809 | 0.2584 | -1.9522 | 0.0161 | 0.178209278 | / |
| 407.28-5.15 | 1.6745 | 1.5813 | 0.6612 | 0.0405 | 0.180019196 | Ursocholic acid |
| 225.11-3.62 | 2.8034 | 0.0940 | -3.4105 | 0.0376 | 0.180052272 | / |
| 817.57-5.15 | 1.9518 | 2.2964 | 1.1994 | 0.0489 | 0.181560765 | / |
| 1223.86-5.15 | 1.7623 | 2.8093 | 1.4902 | 0.0468 | 0.185108813 | alpha-Muricholic acid |
| 144.05-3.55 | 2.9158 | 4.9076 | 2.2950 | 0.0414 | 0.186455555 | / |
| 313.11-4.49 | 1.9369 | 1.5993 | 0.6774 | 0.0371 | 0.186455555 | / |
| 180.07-1.48 | 2.2129 | 3.5978 | 1.8471 | 0.0415 | 0.186455555 | Beta-Tyrosine |
| 457.33-7.18 | 1.9831 | 3.4634 | 1.7922 | 0.0411 | 0.187243771 | / |
| 596.28-4.06 | 1.4058 | 0.0980 | -3.3516 | 0.0187 | 0.187243771 | / |

| | | | | | | |
|---|---|---|---|---|---|---|
| 135.03-0.85 | 2.9528 | 4.8022 | 2.2637 | 0.0297 | 0.187459125 | / |
| 190.05-3.55 | 1.3534 | 4.6865 | 2.2285 | 0.0380 | 0.187459125 | Indole-3-carboxaldehyde |
| 173.08-3.79 | 1.8629 | 1.5410 | 0.6239 | 0.0165 | 0.191395782 | / |
| 533.35-6.08 | 1.2096 | 0.2867 | -1.8026 | 0.0163 | 0.19300759 | / |
| 471.35-6.09 | 2.8434 | 0.2148 | -2.2191 | 0.0024 | 0.195063886 | / |
| 453.34-6.09 | 1.4219 | 0.2176 | -2.2006 | 0.0018 | 0.195063886 | / |
| 705.4-5.13 | 1.7872 | 2.4813 | 1.3111 | 0.0005 | 0.200651618 | / |
| 335.15-3.31 | 1.0784 | 0.2132 | -2.2299 | 0.0243 | 0.206748837 | / |
| 487.31-4.77 | 1.0065 | 2.3678 | 1.2435 | 0.0157 | 0.215732968 | / |
| 483.31-7.07 | 1.4207 | 0.2237 | -2.1604 | 0.0246 | 0.219980846 | / |
| 489.36-6.09 | 1.2143 | 0.2116 | -2.2405 | 0.0060 | 0.222119992 | / |
| 439.36-6.7 | 1.2099 | 0.4708 | -1.0869 | 0.0046 | 0.222372823 | / |
| 269.05-5.02 | 2.1190 | 0.4039 | -1.3080 | 0.0435 | 0.226080404 | 1,2,8-Trihydroxy-3-methylanthraquinone |
| 245.14-4.17 | 1.7702 | 0.5382 | -0.8939 | 0.0080 | 0.227867533 | / |
| 495.3-3.25 | 6.1000 | 0.0876 | -3.5127 | 0.0081 | 0.236500598 | / |
| 134.06-3.9 | 2.1737 | 5.2680 | 2.3973 | 0.0004 | 0.236500598 | / |
| 162.06-3.16 | 2.3720 | 1.2850 | 0.3618 | 0.0063 | 0.24248186 | / |
| 539.29-3.28 | 1.6265 | 0.1113 | -3.1671 | 0.0445 | 0.244462104 | / |

**Table S16.** Statistical parameters of differential metabolites in feces (AGPH vs. Model)

| M/Z-RT (min) | VIP | FC(M) | log2(M) | P-VALUE | FDR | Metabolites |
|---|---|---|---|---|---|---|
| 270.32-6.93 | 4.1824 | 0.8308 | -0.2674 | 0.0002 | 0.0005219 | / |
| 146.12-1.27 | 2.0742 | 2.5353 | 1.3422 | 0.0039 | 0.02999320 | / |

| | | | | | | |
|---|---|---|---|---|---|---|
| | | | | | 8 | |
| 160.13-1.9 | 1.1545 | 2.2804 | 1.1893 | 0.0076 | 0.045231472 | / |
| 316.28-6.11 | 2.0656 | 0.3686 | -1.4399 | 0.0015 | 0.065407783 | / |
| 375.29-6.13 | 1.8713 | 2.6396 | 1.4003 | 0.0258 | 0.124575127 | / |
| 357.28-6.12 | 2.1398 | 2.7148 | 1.4409 | 0.0283 | 0.129252725 | / |
| 391.29-6.11 | 2.7319 | 2.8978 | 1.5350 | 0.0098 | 0.130067366 | / |
| 437.29-6.11 | 2.3641 | 2.7216 | 1.4444 | 0.0046 | 0.130067366 | / |
| 453.29-4.78 | 1.4388 | 2.3441 | 1.2291 | 0.0006 | 0.13722912 | 11-Hydroxyeicosatetraenoate glyceryl ester |
| 333.26-5.27 | 2.7229 | 3.9104 | 1.9673 | 0.0029 | 0.138763753 | / |
| 438.26-7.54 | 2.9584 | 0.5912 | -0.7583 | 0.0034 | 0.151018057 | / |
| 315.12-4.08 | 2.6844 | 1.4223 | 0.5082 | 0.0132 | 0.151430385 | / |
| 357.28-5.96 | 4.1388 | 2.9634 | 1.5672 | 0.0143 | 0.153903445 | / |
| 357.28-6.57 | 2.2372 | 1.9787 | 0.9846 | 0.0484 | 0.156065936 | / |
| 228.05-2.45 | 2.9173 | 0.1351 | -2.8879 | 0.0045 | 0.159663641 | pipecolic acid betaine |
| 437.29-5.95 | 4.7156 | 2.3269 | 1.2184 | 0.0048 | 0.164807571 | Isodeoxycholic acid |
| 571.29-7.74 | 2.0511 | 0.2284 | -2.1302 | 0.0027 | 0.164807571 | / |
| 243.12-3.65 | 2.6520 | 1.8090 | 0.8552 | 0.0110 | 0.169445879 | / |
| 243.12-3.73 | 1.2335 | 2.0598 | 1.0425 | 0.0034 | 0.170498377 | / |
| 375.29-6.57 | 1.8276 | 1.9408 | 0.9567 | 0.0248 | 0.172659029 | / |
| 571.29-7.87 | 1.5698 | 0.0076 | -7.0311 | 0.0002 | 0.174337431 | / |
| 391.29-6.56 | 2.7205 | 1.9193 | 0.9406 | 0.0498 | 0.181419841 | / |

| | | | | | | |
|---|---|---|---|---|---|---|
| 226.04-2.46 | 2.7037 | 0.1320 | -2.9210 | 0.0025 | 0.182032559 | Quinolinic acid |
| 245.14-4.17 | 2.2542 | 0.4908 | -1.0269 | 0.0080 | 0.18272918 | / |
| 285.17-3.61 | 1.5575 | 1.4096 | 0.4953 | 0.0161 | 0.18272918 | / |
| 183.03-2.46 | 1.9583 | 0.1318 | -2.9238 | 0.0031 | 0.183279862 | / |
| 361.22-3.75 | 1.7316 | 1.3537 | 0.4369 | 0.0089 | 0.185613263 | / |
| 285.11-4.2 | 1.9972 | 1.8471 | 0.8852 | 0.0284 | 0.186315362 | / |
| 389.27-4.57 | 1.1412 | 1.5617 | 0.6432 | 0.0157 | 0.188310231 | 14-HDoHE |
| 274.09-0.84 | 1.2862 | 0.3154 | -1.6649 | 0.0415 | 0.188854643 | / |
| 243.12-3.55 | 5.8255 | 1.8508 | 0.8882 | 0.0165 | 0.190149817 | 10-hydroxydodec-5-enedioic acid |
| 783.58-5.95 | 2.1522 | 6.4916 | 2.6986 | 0.0050 | 0.190149817 | 3beta,12beta-Dihydroxy-5beta-cholanoic acid |
| 454.35-5.78 | 1.1958 | 0.3367 | -1.5705 | 0.0206 | 0.204406428 | / |
| 134.06-3.9 | 3.0228 | 5.7890 | 2.5333 | 0.0004 | 0.207254817 | / |
| 329.1-4.21 | 5.3624 | 1.9730 | 0.9804 | 0.0138 | 0.211901096 | / |
| 229.14-5.31 | 2.0582 | 3.7886 | 1.9217 | 0.0021 | 0.2224029 | / |
| 487.31-4.77 | 1.6097 | 2.5434 | 1.3467 | 0.0157 | 0.224969278 | / |
| 575.27-3.88 | 1.6321 | 0.2533 | -1.9813 | 0.0124 | 0.226232587 | / |
| 377.12-3.29 | 2.2479 | 1.7458 | 0.8039 | 0.0197 | 0.226917086 | / |
| 270.32-7.24 | 5.3954 | 1.7446 | 0.8029 | 0.0057 | 0.231250254 | / |
| 245.14-3.55 | 1.6879 | 1.8943 | 0.9217 | 0.0300 | 0.231250254 | / |
| 163.11-3.55 | 1.1409 | 1.7979 | 0.8463 | 0.0348 | 0.231253918 | / |
| 121.1-3.55 | 1.3120 | 1.8239 | 0.8670 | 0.0306 | 0.241532342 | / |
| 329.1-4.14 | 2.8218 | 1.9683 | 0.9769 | 0.0373 | 0.242323489 | / |

| | | | | | | |
|---|---|---|---|---|---|---|
| 290.09-1.29 | 1.4588 | 0.3655 | -1.4522 | 0.0406 | 0.24710645<br>9 | / |
